# Identification of differential microRNA expression during tooth morphogenesis in the heterodont dentition of miniature pigs, SusScrofa

**DOI:** 10.1186/s12861-015-0099-0

**Published:** 2015-12-29

**Authors:** Ang Li, Ye Li, Tieli Song, Fu Wang, Dayong Liu, Zhipeng Fan, San Cheng, Chunmei Zhang, Jinsong Wang, Junqi He, Songlin Wang

**Affiliations:** Molecular Laboratory for Gene Therapy and Tooth Regeneration, Beijing Key Laboratory of Tooth Regeneration and Function Reconstruction, Capital Medical University School of Stomatology, Tian Tan Xi Li No.4, Beijing, 100050 China; Key Laboratory of Shaanxi Province for Craniofacial Precision Medicine Research, College of Stomatology, Xi’an Jiaotong University, Xi Wu Lu No.98, Xi’an, 710004 China; Laboratory of Molecular Signaling and Stem Cells Therapy, Beijing Key Laboratory of Tooth Regeneration and Function Reconstruction, School of Stomatology, Capital Medical University, Tian Tan Xi Li No.4, Beijing, 100050 China; Department of Biochemistry and Molecular Biology, Capital Medical University School of Basic Medical Sciences, You An Men Wai Xi TouTiao No.10, Beijing, 100069 China

**Keywords:** Odontogenesis, Morphogenesis, Microarray analysis, *In situ* hybridization, Miniature swine

## Abstract

**Background:**

It has been found that microRNAs (miRNAs) play important roles in the regulation of tooth development, and most likely increase the complexity of the genetic network, thus lead to greater complexity of teeth. But there has been no research about the key microRNAs associated with tooth morphogenesis based on miRNAs expression profiles. Compared to mice, the pig model has plentiful types of teeth, which is similar with the human dental pattern. Therefore, we used miniature pigs as large-animal models to investigate differentially expressed miRNAs expression during tooth morphogenesis in the early developmental stages of tooth germ.

**Results:**

A custom-designed miRNA microarray with 742 miRNA gene probes was used to analyze the expression profiles of four types of teeth at three stages of tooth development. Of the 591 detectable miRNA transcripts, 212 miRNAs were continuously expressed in all types of tooth germ, but the numbers of miRNA transcript among the four different types of teeth at each embryonic stage were statistically significant differences (*p* < 0.01). The hierarchical clustering and principal component analysis results suggest that the miRNA expression was globally altered by types and temporal changes. By clustering analysis, we predicted 11 unique miRNA sequences that belong to mir-103 and mir-107, mir-133a and mir-133b, and mir-127 isomiR families. The results of real-time reverse-transcriptase PCR and *in situ* hybridization experiments revealed that five representative miRNAs may play important roles during different developmental stages of the incisor, canine, biscuspid, and molar, respectively.

**Conclusions:**

The present study indicated that these five miRNAs, including ssc-miR-103 and ssc-miR-107, ssc-miR-133a and ssc-miR-133b, and ssc-miR-127, may play key regulatory roles in different types of teeth during different stages and thus may play critical roles in tooth morphogenesis during early development in miniature pigs.

**Electronic supplementary material:**

The online version of this article (doi:10.1186/s12861-015-0099-0) contains supplementary material, which is available to authorized users.

## Background

Many studies have revealed that microRNAs (miRNAs) play important roles in the regulation of tooth development. Indeed, during evolution, there has been a decrease in tooth number and an increase in tooth complexity, which may reflect changes in the balance of genetic networks and network robustness. MiRNAs have most likely increased the complexity of the genetic network and thus led to greater complexity of teeth.

Three functional studies had used deletion of dicer-1 (a protein involved in the maturation of miRNA) to analyze miRNA function during tooth development. Conditional deletion of dicer-1 in the epithelium results in mild but significant aberrations in tooth shape and enamel formation [1]. Dicer-1 deletion in another study also resulted in multiple and branch enamel-free incisors and cuspless molars, as well as changes in incisor patterning, incisor and molar size and shape [2]. A recent study in mice with a conditional deletion of dicer-1 in the mesenchyme showed an arrest or absence of tooth development between the incisors and molars; moreover, extra incisor tooth formation was found in the dicer-1 deletion epithelium [3].

Using miRNA expression profiling, many studies have found that differentially expressed miRNAs are expressed during tooth development. For example, hsa-miR-133a, hsa-miR-200b, hsa-miR-206, and hsa-miR-218 were considered as tooth tissue-specific miRNAs [4]; eight differentially expressed miRNAs were expressed during morphogenesis and seven were expressed in the incisor cervical loop containing the stem cell niche [1]; the three most highly expressed microRNAs in dental epithelium were identified as mmu-miR-24, mmu-miR-200c, and mmu-miR-205, while mmu-miR-199a-3p and mmu-miR-705 were found in dental mesenchyme [2]; and miR-200 was suggested to play an important role in the formation of incisor cervical loop during stem cell–fueled incisor growth [5]. But there was no research about the key microRNAs associated with tooth morphogenesis based on miRNAs expression profiles.

All of the studies mentioned above were performed in mice, which are good animal models used to study organ development. However, mouse teeth are different from those of humans in both number and morphology. More importantly, mice lack the full spectrum of teeth; there are only one incisor and three molars in each region of the mouse dentition. By contrast, humans have four types of teeth: incisors, canines, premolars, and molars. As a large animal species, pig (e.g., miniature pigs, also known as minipigs) teeth and jawbones are similar to those of humans [6]. Pig deciduous teeth share numerous morphological similarities with human dental pattern. Thus, pigs are considered to be suitable models used to conduct tooth development studies, especially in regard to tooth morphogenesis.

However, little information is available regarding to the miRNA regulatory processes that occur during tooth development in pigs [7]. In the present study, we used minipigs as a large animal model to investigate key miRNA expression in heterodont dentition morphogenesis during the early developmental stages of tooth germ.

## Methods

### Animals and sample preparation

The Wuzhishanminipigs used in the experiments were obtained from the Kexing Laboratory Animal Company of Beijing, China. Experiments were performed according to the Regulations for the Administration of Affairs Concerning Experimental Animals and were approved by the Animal Care and Use Committees of Capital Medical University (Beijing, China) under permit number CMU-B20100106. The staged miniature pig embryos and fetuses were obtained by cesarean section at embryonic day 40 (E40), E50, and E60; these stages cover the major morphological and physiological changes in pig tooth germ growth and development throughout pregnancy, including the cap, early bell, and late bell stages [8–10].

After surgically removing the fetuses, germ tissue samples from four different types of teeth were removed from the mandibles under microscope: first deciduous incisor (Di), deciduous canine (Dc), second deciduous premolar (Dpm), and deciduous molar (Dm). The liver, kidney and submandibular gland tissues coming from the same source were also saved as control samples. The samples were immediately frozen in liquid nitrogen and stored at −80 °C. Samples from each pregnant pig were kept separately. Specimens for histological study were chosen by random selection from the four different types of teeth from fetuses in the three developmental stage groups.

### MiRNA extraction and custom-designed miRNA microarray screening

We confirmed 742 miRNA gene probes using custom-designed miRNA microarrays [7]. To ensure stability, probe assays were performed in quadruplicate. We carried out a comparative miRNA expression profile analysis across 12 tooth germ samples independently collected from pig embryos: Di40, Di50, Di60, Dc40, Dc50, Dc60, Dpm40, Dpm50, Dpm60, Dm40, Dm50, and Dm60 (Additional file 1). Total RNA was isolated using an RNA purification kit (Illumina, San Diego, USA) according to the manufacturer’s instructions, and then checked for integrity using an Agilent Bioanalyzer 2100 (Agilent technologies, Santa Clara, CA, USA).

The microarray-expression assay was performed by a service provider (LC Sciences, Houston, Texas, USA). Briefly, 5 μg RNA from each developmental-stage sample was size fractionated (<300 nucleotides) with YM-100 microcon centrifugal filter (Millipore, Billerica, MA). Hybridization was performed overnight on a μParaflo microfluidic chip using a microcirculation pump (Atactic Technologies, Houston, TX). After RNA hybridization, tag-conjugating Cy3 and Cy5 dyes were circulated through the microfluidic chip for dye staining. Fluorescence images were collected using a laser scanner (GenePix 4000B, Silicon Valley, CA) and digitized using Array-Pro image analysis software (Media Cybernetics, Bethesda, MD). Pairs of labeled samples from different stages were hybridized to dual-channel microarrays. The microarray data meet MIAME requirements and have been deposited in the publicly available database Gene Expression Omnibus (GEO; http://www.ncbi.nlm.nih.gov/projects/geo/, GSE53660).

### In depth analysis of miRNA microarray data

Firstly, we used lowess methods [11] to normalize the microarray data, then carried out a hierarchical clustering approach to detect clusters of miRNAs and 12 tooth germ samples. In detail, we applied bottom up complete linkage clustering and used the WGCNA measure, an R package for weighted correlation network analysis (http://labs.genetics.ucla.edu/horvath/CoexpressionNetwork/Rpackages/WGCNA/). In addition, we carried out a standard principal component analysis (PCA) based on perl script, provide scatter plots of the first versus second versus third principal component [12] and then drew figure by using R method (http://www.r-project.org/).

We also analyzied differentially expressed miRNAs of samples using miRNA expression patterns by applying t-test, ANOVA and clustering analysis methods. Briefly, we compared the miRNAs expression profiles of four type teeth during three stages based on the ANOVA, then pair-wisely compared every type tooth during three stages based on t-test. In detail, the miRNAs with lowest *p*-values (*P* < 0.05 or 0.01) , significant differential expression (a fold change >2) and strong signals intensity (signal ≥500) were considered to be candidate differentially expressed miRNAs.

### Quantitative real-time RT-PCR

To validate the microarray data, we used a miRNA quantification method similar to that described previously [13]. To ensure the accuracy, three biological replicates were performed (three pregnant sows per time point include E40, E50 and E60). DNAStar version 6.1.3 software was used to design the primer sequences, which were listed in Additional file 2. Firstly, 100 ng of total RNA was reverse transcribed using 100 U of M-MLV reverse transcriptase (RT) and 1 ſM stem-loop RT primer in a 7900 Thermocycler (Applied Biosystems, Carlsbad, CA, USA) with incubation at 42 °C for 60 min and 70 °C for 15 min. The samples were then kept at 4 °C. Real-time PCR was performed using Platinum SYBR Green qPCRSuperMix-UDG. Porcine ssc-miR-24 [14] was used as an internal control. All samples were analyzed in triplicate and the expression of target genes was calculated as relative fold values using the 2^−△△CT^ method. The significance level was set to *p* < 0.05. Pearson’s correlation coefficient was further calculated for each gene using the normalized data to quantify the consistency between microarray experiments and qRT-PCR (*p <* 0.05 and *R* > 0.9).

### *In situ* hybridization

Embryonic mandibles were fixed in 4 % paraformaldehyde and processed for paraffin sections as previously described [15]. Coronal paraffin sections of 6 μm were processed for locked nucleic acid–digoxigenin (LNA–DIG) *in situ* hybridization as previously described [16]. A scramble probe was used as negative control, as it was representative of a nonspecific positive signal. All of the LNA probe sequences that we used can be found out on the Exiqon website (www.exiqon.com) or in Additional file 3.

## Results

### MiRNA expression patterns in different tooth types during tooth development

Of the 742 miRNA probes, only 151 had no detectable signal in any of the samples. A total of 212 miRNAs were ubiquitously expressed in all types of tooth germ during all developmental stages (Additional file 4). These results indicate that the chip we designed was able to specifically detect pig miRNAs and suggest that many pig-specific miRNAs may play functional roles in tooth germ development.

**Fig. 1:**
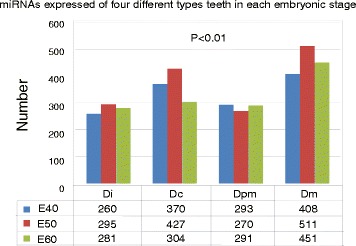
Number of MiRNAs expressed in each type of tooth. Of the detectable miRNA transcripts, 260–295 were expressed in the first deciduous incisor (Di), 304–427 were expressed in the deciduous canine (Dc), 270–291 were expressed in the second deciduous premolar (Dpm), and 408–511 were expressed in the deciduous molar (Dm) from E40 to E60. Differences in the number of miRNAs expressed at each stage were statistically significant (*p* = 0.0024)

Of the 591 detectable miRNA transcripts, an average of 279 were expressed in Di, 367 in Dc, 285 in Dpm, and 457 in Dm (Additional file 4). The differences in the numbers of miRNAs expressed in the four different types teeth at each embryonic stage werestatistically significant (*p* < 0.01) (Fig. 1). There was a correlation between the complexity of the tooth morphology and the number of miRNAs involved in regulation. These results suggest that miRNAs may play an important role in tooth morphogenesis.

### Hierarchical clustering analysis and principal component analysis

**Fig. 2:**
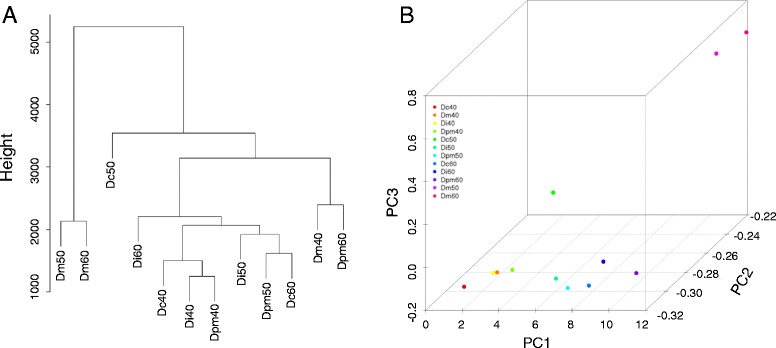
Cluster analysis and Principal Component Analysis of all tooth germ samples. **a** Cluster dendrogram of tooth germ samples from different types and from different time points of the test and validation set. **b** Principal Component Analysis of tooth germ samples from different types and from different time points of the test and validation set. The figure shows the first (*x-axis*) versus the second (*y-axis*) versus the third (*z-axis*) principal component. The samples were analyzed according to the expressions of 591 detectable miRNAs using auto-scale method. First deciduous incisor (Di), deciduous canine (Dc), second deciduous premolar (Dpm), and deciduous molar (Dm). The miRNA expression was globally altered by types and temporal changes

To globally view the teeth type changes of miRNAs expression at different development time points, hierarchical clustering and principal component analysis were performed to classify all of the 12 tooth germ samples (Di40, Di50, Di60, Dc40, Dc50, Dc60, Dpm40, Dpm50, Dpm60, Dm40, Dm50, and Dm60). The normalized Ct values of 591 detectable miRNAs (Additional file 4) were used for this analysis. As shown in the dendrogram in Fig. 2a, the samples can be roughly divided into four clusters. Splitting the dendrogram in 4 groups and computing a contingency table, we found that all tooth germ samples belong to the different conditions segregate from one another, implying that developmental stage does affect the expression profile in most cases.

To provide a low-dimensional visualization of the high dimensional data we carried out a principal component analysis. A plot of the first versus the second versus the third principal component is shown in Fig. 2b. The principal component analysis largely confirmed the results of the hierarchical clustering. The results once again suggest that miRNA expression was globally altered by types and temporal changes.

### Differentially expressed miRNAs in tooth morphogenesis during tooth early development

**Fig. 3:**
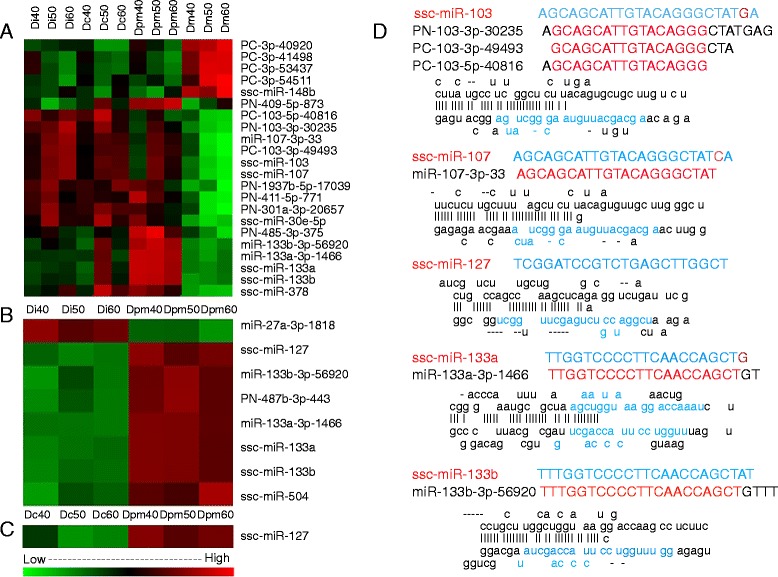
Clustering analysis of key microRNA in four tooth types at different stages. **a** Twenty-two miRNAs were differentially expressed among the four tooth types during E40, E50, and E60, based on cluster analyses (*p* < 0.01). Red indicates a gene highly expressed at this stage, whereas green indicates the opposite. **b**, **c** Pairwise comparisons based on cluster analyses revealed anther eight differentially expressed miRNAs between the first deciduous incisor (Di) and the second deciduous premolar (Dpm) (*p* < 0.01), and one (ssc-miR-127) between the deciduous canine (Dc) and Dpm (*p* < 0.05) during the three developmental stages. **d** Seed sequences and duplexes (boxes) of five isomiR families including 11 miRNAs had high-signal (signal ≥500) miRNA transcripts based on cluster analyses of miRNA expression patterns, thus identifying them as key microRNAs

By clustering analysis, we found 22 miRNAs that were differentially expressed in the 12 samples (*p* < 0.01) (Fig. 3a). Eleven of these had high signals (signal ≥500) (Additional file 5). When we focused upon these miRNAs, we found that most of them were homologous isomers. We then predicted that the miR-103, and miR-107, miR-133a, and miR-133b isomiRs would be differentially expressed miRNAs. PN-1937b-5p-17039 was not selected as a potential key miRNA because there was anomalous peak expression in all samples, which could imply that it probably does not have an important function in a certain type tooth; at the same time, the only homologous isomer of PN-1937b-5p-17039 is mmu-mir-5103, which only exists in mice but the function is unknown.

After performing six pairwise comparisons among the subgroups, we added another candidate miRNA, miR-127, which was significantly differentially expressed among Di, Dc, and Dpm (Fig. 3b–c). In total, our analysis yielded 11 unique miRNA sequences that belong to five isomiR families and may have key roles in tooth development. The seed sequences and duplexes (boxes) of them were showed in Fig. 3d.

### Validation of differentially expressed miRNAs by real-time RT-PCR

**Fig. 4:**
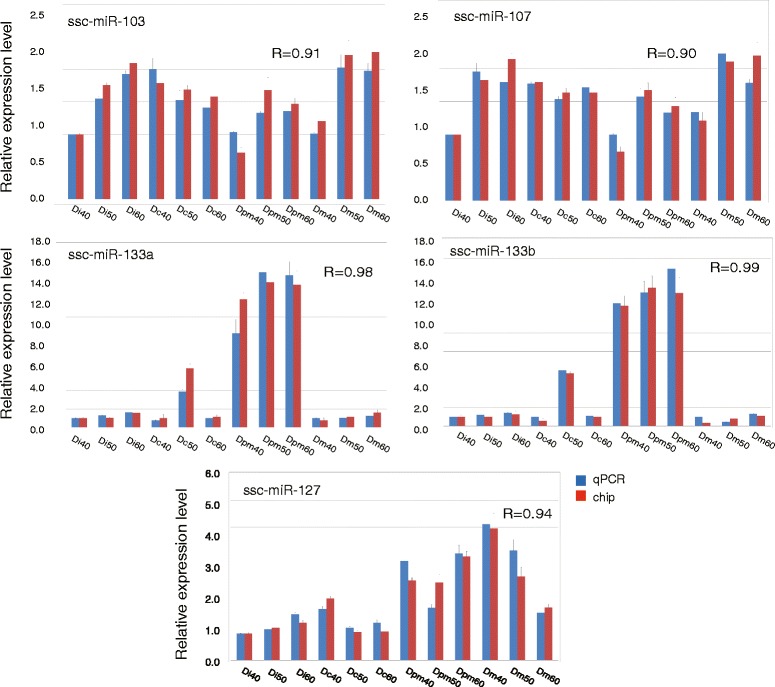
Validation of miRNAs by real-time RT-PCR. Expression levels of five miRNAs (ssc-miR-103, ssc-miR-107, ssc-miR-127, ssc-miR-133a, and ssc-miR-133b) were detected by real-time RT-PCR and microarray chip. We scaled the raw data from the real-time RT-PCR to make it comparable with the microarray data. Each time point was replicated three times using independently collected samples; the average is shown with the standard deviation. *R* represents the Pearson correlation coefficient. Expression levels of the five miRNAs were in concordance with the normalized microarray data (*R* > 0.9). Ssc-miR-103 and ssc-miR-107 expression was slightly lower in premolars (Dpm) than in other types of teeth, ssc-miR-133a and ssc-miR-133b expression was much higher in Dpm than in other types of teeth, and ssc-miR-127 expression gradually increased from the incisor (Di) to the molar (Dm)

The five representative sus-scrofa’s miRNAs described above were validated by real-time RT-PCR using 12 independent samples. Expression levels of all of the selected miRNAs were in concordance with the normalized microarray data (Pearson correlation coefficient >0.9, Fig. 4). In general, the results of the real-time RT-PCR validated the accuracy of the microarray. We also found that expression levels of ssc-miR-103 and ssc-miR-107 were slightly lower in Dpm than in other types of teeth, ssc-miR-133 a and ssc-miR-133b expression levels were much higher in Dpm than in other types of teeth, and ssc-miR-127 expression increased in Di, Dc, Dpm, and Dm, in that order. A schematic diagram (Fig. 4) was created that placed these miRNAs into three possible expression pattern profiles among the three developmental stages.

**Fig. 5:**
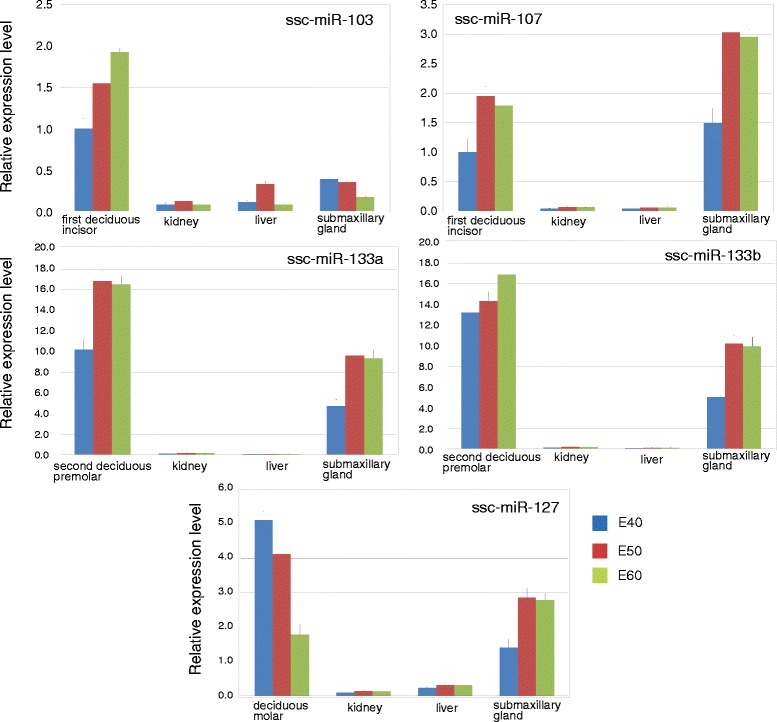
The expression levels of five selected miRNAs in developing primary deciduous teeth compared with kidney, liver and submandibular gland in E40, E50 and E60. Real-time RT-PCR results showed the changes in expression levels of four different tissues. The data are a representative of three independent experiments. *P value* < 0.05

In order to detect the oral developmental specificity of the five selected miRNAs, we further extracted kidney, liver and submandibular gland to contrast the five miRNAs’ expression (Fig. 5). Kidney and liver were chosen on behalf of the tissues of non-oral-maxillofacial development and submandibular gland as the representative of oral-maxillofacial development tissue. For ssc-miR-103 and ssc-miR-107, we chose the first deciduous incisor to contrast with the three kinds of tissues. We could found that the expression levels of ssc-miR-103 were strongly lower in kidney, liver and submandibular gland, while the expression levels of ssc-miR-107 were strongly lower in kidney and liver but somehow relatively high in submandibular gland. For ssc-miR-133a and ssc-miR-133b, we chose the second deciduous premolar to contrast with the three kinds of tissues. The expression levels of both two miRNAs in kidney and liver were negligible compare with the second deciduous premolar and submandibular gland. For ssc-miR-127, deciduous molar were chose as the reference, the expression level is fairly higher in deciduous molar and submandibular gland compared to that in kidney and liver.

### Localization of differentially expressed miRNAs in tooth morphogenesis during tooth early development

We next localized expression of the five selected miRNAs by *in situ* hybridization in the germs of Di, Dc, Dpm, and Dm from stages E40, E50, and E60 (Table 1). The overall trend in miRNA expression detected in the tissue sections was consistent withthe microarray and qRT-PCR results, but there were some variations in expression among the different stages of tooth development.

**Table 1:** Expression level of 5 differentially expressed miRNAs in four type teeth during three tooth developmental stages

			ssc-miR-103	ssc-miR-107	ssc-miR-127	ssc-miR-133a	ssc-miR-133b
Incisor (Di)	E40	Epithelium	**+++**	++**+**	+	++	++
		Mesenchyme	+++	++**+**	+	++	++
	E50	Epithelium	++	++	+	+	+
		Mesenchyme	++	++	+	+	+
	E60	Epithelium	++	+++	++	+	+
		Mesenchyme	+	+	+	+	+
Canine (Dc)	E40	Epithelium	+++	++	+	+	+
		Mesenchyme	+++	++	+	+	+
	E50	Epithelium	++	++	+	+	+
		Mesenchyme	+	++	+	+	+
	E60	Epithelium	++	++	++	+	+
		Mesenchyme	+	+	+	+	+
Premolar (Dpm)	E40	Epithelium	-	-	+	+	+
		Mesenchyme	-	-	+	+	+
	E50	Epithelium	-	-	-	+	+
		Mesenchyme	-	-	-	+	+
	E60	Epithelium	++	++	++	+++	+++
		Mesenchyme	+	+	+	+++	+++
Molar (Dm)	E40	Epithelium	++	+++	+++	+	+
		Mesenchyme	+	++	+++	+	+
	E50	Epithelium	+++	+++	++	+	++
		Mesenchyme	++	++	+	+	+
	E60	Epithelium	+++	+++	-	++	++
		Mesenchyme	++	++	-	+	+

-, negative; +, low; ++, medium; and +++, high level of expression. The level of expression was assessed of each probe separately

**Fig. 6:**
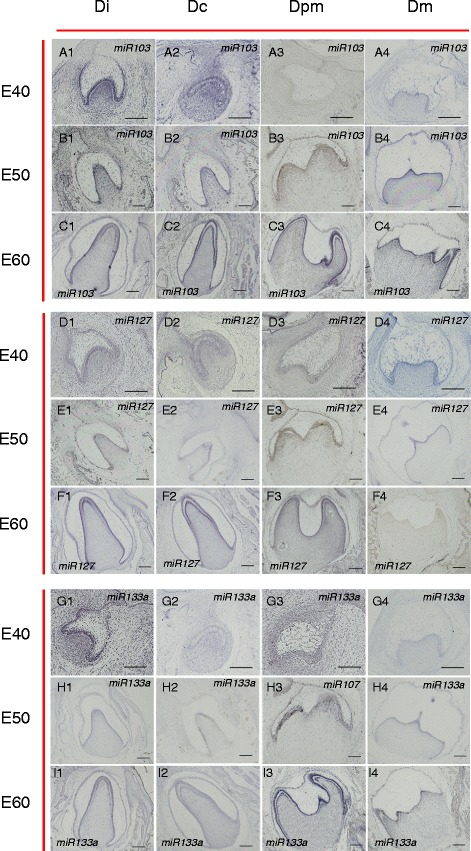
In situ hybridization revealed expression patterns of miRNAs in four types of teeth during three tooth developmental stages. (A1–C4) At E40, miR-103 was expressed in both the epithelium and mesenchyme of the incisor, canine, and molar, but expression in the premolar was lower and expression in the molar was not as strong as in the incisor and canine (A1–A4). At E50, miR-103 expression in all four types of teeth stayed the same for the most part, but the expression level was lower and more restricted in the inner enamel epithelium, which contains the most important structure of morphogenesis: the enamel knot in incisor and canine. Expression in the molar was increased and restricted in the inner enamel epithelium as well (B1–B4). At E60, mir-103 expression in the premolar increased substantially, while in the other three types of teeth, the location was restricted in the inner enamel epithelium (C1–C4). (D1–F4) At E40, miR-127 was expressed in both the epithelium and mesenchyme of the incisor, canine, premolar, and molar, but the expression levels among the four types of teeth were different. There was a strong signal in the molar, in contrast to a weak signal in the other three types of teeth (D1–D4). At E50, miR-127 expression in all four types of teeth stayed almost the same, but expression in the premolar was not observed and the expression level was lower and more restricted in the inner enamel epithelium of the molar (E1–E4). At E60, mir-127 expression in the incisor, canine, and premolar increased, while expression decreased in the molar. The location of mir-127 was restricted in the inner enamel epithelium of the incisor, canine, and premolar (F1–F4). (G1–I4) At E40, miR-133a was expressed in both the epithelium and mesenchyme of all four types of teeth, with a higher signal in the incisor (G1–G4). At E50, miR-133a expression in all four types of teeth stayed nearly the same, but with a lower signal in the incisor (H1–H4). At E60, expression was more restricted in the inner enamel epithelium and increased expression was found in the premolar and molar (I1–I4). Scale bar, 200 μm. Di, first deciduous incisor; Dc, deciduous canine; Dpm, second deciduous premolar; Dm, deciduous molar; E40, embryonic day 40; E50, embryonic day 50; E60, embryonic day 60

All the five selected miRNAs were expressed in four types of teeth in miniature pigs, both in epithelium and mesenchyme. Ssc-mir-103 was located in the outer and inner enamel epithelium, dental mesenchyme, and stellate reticulum (Fig. 6A1–C4). Ssc-mir-107 expression was similar to that of ssc-miR-103 (Additional file 6A1–C4). These results suggest that ssc-miR-103 and ssc-miR-107 may play important roles in the early morphogenesis of conoides teeth and in crosstalk between epithelial and mesenchymal tissue.

Ssc-mir-127 *in situ* expression reflected the microarray and real-time RT-PCR results (Fig. 6D1–F4). It was also located in the outer and inner enamel epithelium, dental mesenchyme, and stellate reticulum. But the expression levels in epithelium and mesenchyme of different types of teeth differed. In combination with our other results, this implies that ssc-miR-127, ssc-miR-103, and ssc-miR-107 may play a regulatory role in the morphogenesis of all kinds of teeth during different developmental stages.

Both ssc-miR-133a (Fig. 6G1–I4) and ssc-miR-133b (Additional file 6D1–F4) were strongly expressed in the epithelium and mesenchyme of Dpm, in contrast with the other three potentially differentially expressed miRNAs. This suggests that ssc-miR-133a and ssc-miR-133b may play more important roles in the early morphogenesis of premolar. Validation of differentially expressed miRNAs detection in both epithelium and mesenchyme by real-time RT-PCR was performed and shown in Additional file 7.

## Discussion

Despite differences in the final shape, teeth undergo successive developmental stages that are common to all mammals, including epithelial thickening, bud, cap and bell stages. The increase in tooth complexity is reflected in the patterning of cusps which develop from the initiation of cap stage. The shapes of all minipig tooth anlagen are similar at early stages of development (from the initiation of the cap stage to the late bell stages). Differences become obvious at the beginning of the late bell stage, when characteristic shaping of the inner enamel epithelium occurs [9]. Thus, we selected the cap (E40), early bell (E50), and late bell (E60) stages of tooth germ for inclusion in our study.

The minipig is an excellent experimental model for tooth morphogenesis because its heterodont dentition resembles that of humans. The dental formula for deciduous dentition of the miniature pig is i3/3, c1/1, and p3/3 = 28. However, based on crown morphology, we thought that it would be appropriate to consider the third deciduous premolar as a molar, which is consistent with the perspective of Bivin and McClure [8] but different from that of Stembirek et al. [9] and Wang et al. [17]. Importantly, the four different types of teeth included in our study (i.e., Di, Dc, Dpm, and Dm) are at close to the same developmental stage (cap, early bell, and late bell) at the same time points (E40, E50, and E60) [17]. These morphological changes that take place during these stages facilitate analysis of pattern formation in heterodont dentition that can potentially be used to address hypotheses concerning the morphogenetic specificity of the incisor, canine, premolar, and molar areas.

To view whether stage or tooth type affects the miRNAs expression profiles, we performed cluster and principal component analysis. The bioinformatics analysis results suggest that the miRNA expression was globally altered by types and temporal changes. To identify the specificity of the miRNAs in tooth development, we contrast the five miRNAs’ expressions between dental and non-dental tissues by qRT-PCR. All the expression levels of five miRNAs were fairly high in tooth except ssc-miR-107, and relatively high in submandibular gland except ssc-miR-103, and lower in kidney and liver. These results suggest that craniofacial organogenesis including tooth and salivary gland not only develops in a similar regulatory mechanism pattern during development [4], but also have some similar miRNAs.

Of the five differentially expressed miRNAs that we identified, miR-133 (miR-133a and miR-133b), which is specifically expressed in muscles, is classified as a myomiRNA and is necessary for proper skeletal and cardiac muscle development and function [18]. MiR-133 is one of tissue-specific miRNAs in tooth germ [4], and in Michon’s miRTooth1.0 Database (http://bite-it.helsinki.fi/), it is described as being specifically expressed at the cap, bell, and differentiation stages of tooth germ in mouse [19]. In another study, mmu-miR-133a and mmu-miR-133b were found to be highly expressed at E13.5 in the mouse molar [3]. We also suggested in a previous study that ssc-miR-133 may play key roles in miniature pigs’s tooth development [7]. Combined with the results of our current study, which showed that these two isomiRs are distinctly expressed in Dpm during E60 (late bell stage), we have reason to believe that ssc-miR-133a and ssc-miR-133b may be differentially expressed miRNAs in multiple pathways involved in bicuspid teeth morphogenesis.

Previous studies have demonstrated that miR-103 and miR-107 may regulate human metabolic pathways that involve cellular acetyl-CoA and lipid levels [20]. Although the miRTooth1.0 Database does not report on the expression of these two miRNAs at specific stages or in specific types of teeth, they do appear to be expressed during most periods of tooth development. In our study, they were also broadly expressed in all types of teeth at nearly every stage, but the complete lack of expression of ssc-miR-103 and ssc-miR-107 in Dpm during E40 and E50 is worthy of attention, as this could indicate that they exist in bidirectional antagonism with ssc-miR-133a and ssc-miR-133b during premolar morphogenesis in large animal species.

MiR-127 is an important regulator of MMP-13 in human chondrocytes and may contribute to the development of osteoarthritis [21]. It is also considered to be associated with the transcription factor Pitx in the miRTooth1.0 Database. In our study, its high expression in Dm during E40 and completely lack of expression in E60 strongly suggests it may be a key miRNA regulator of molar morphogenesis in large animal species.

MiRNAs are believed to be important in tooth morphogenesis and differentiation via their regulation of gene expression, but little is known of their mechanisms and functions in tooth morphogenesis and differentiation. Using injections of anti-mmu-miR-214 into the mandible close to the developing first molar in newborn mice resulted in hypomineralization of the enamel with remnants of organic material and reduced surface roughness after acid etching [22]. Levels of expression of Clu and Tgfb1 in teeth are markedly decreased following in vivo transfection with anti-mmu-miR-214 [23]. The role of a novel Pitx2: miR-200c/141: noggin regulatory pathway in dental epithelial cell differentiation has also been described [24]. Mmu-miR-135a ectopically overexpressed with a lentivirus in tooth germ during the cap stage, revealed that Bmp signaling, specifically Bmpr-Ia and Bmpr-Ib, regulates tooth formation in conjunction with this miRNA [25]. Moreover, Tbx1 had been confirmed to regulate the proliferation of dental progenitor cells and craniofacial development through miR-96-5p and PITX2 [26]. The results of our study may be of use to researchers designing hypothesis-driven functional studies of these miRNAs. In the future, we plan to inhibit expression or overexpress the five differentially expressed miRNAs identified in the present study both in vivo and in vitro to determine their functions in tooth morphogenesis and differentiation.

## Conclusions

In the present study, we showed that 212 miRNAs were ubiquitously expressed in all types of tooth germ in swine, although there were significant differences in the numbers of miRNAs expressed in the four different types of teeth at each embryonic stage. Microarray, real-time RT-PCR, and *in situ* hybridization experiments revealed that ssc-miR-103 and ssc-miR-107, ssc-miR-133a and ssc-miR-133b, and ssc-miR-127 may play more important roles in Di and Dc, Dpm, and Dm, respectively, during different developmental stages. Thus, these five miRNAs may be differentially expressed miRNAs in the development of specific tooth types during different stages.

## Availability of supporting data

The data sets supporting the results of this article are included within the article and its additional files.

## Additional files

Additional file 1:
**Sample sets information in this study.** (DOC 71 kb) Additional file 2:
**Primer sequences of the real-time PCR experiments.** (DOC 72 kb) Additional file 3:
**LNA Probe sequences of five miRNAs.** (DOC 30 kb) Additional file 4:
**Detectable transcripts in different type tooth germ during different development stages.** (DOC 1429 kb) Additional file 5:
**Statistic Test and Clustering Analysis of key microRNA.** (DOC 108 kb) Additional file 6:
**Expression patterns of miRNAs in the four types of teeth during three tooth developmental stages revealed by in situ hybridization.** (A1–C4) At E40, miR-107 was expressed in both the epithelium and mesenchyme of the incisor, canine and molar, but expression in the premolar was not detected by in situ hybridization (A1–A4). At E50, localization of miR-107 in all four types of teeth stayed the same, but the expression level was more restricted in the inner enamel epithelium in the incisor, canine, and molar (B1–B4). At E60, mir-107 expression in the premolar increased significantly; in the premolar as in the other three types of teeth, the location was restricted in the inner enamel epithelium (C1–C4). (D1–F4) At E40, miR-133b was expressed in the both epithelium and mesenchyme of all four types of teeth, with a higher signal in the incisor and a lower signal in the other three types of teeth (D1–D4). At E50, miR-133b expression in all four types of teeth stayed the same, but with a lower signal in the incisor (E1–E4). At E60, expression was more restricted in the inner enamel epithelium and increased expression was found in the premolar and molar (F1–F4). Scale bar, 200 μm. Di, first deciduous incisor; Dc, deciduous canine; Dpm, second deciduous premolar; Dm, deciduous molar; E40, embryonic day 40; E50, embryonic day 50; E60, embryonic day 60. (PDF 4755 kb) Additional file 7: Validation of differentially expressed miRNAs detection in both epithelium and mesenchyme by real-time RT-PCR. We separated epithelium and mesenchyme from each tooth germ and detected the expression of each miRNA respectively. Each detection was replicated three times and the average is shown with the standard deviation. (PDF 4386 kb)

## Abbreviations

E: embryonic day
Di: first deciduous incisor
Dc: deciduous canine
Dpm: second deciduous premolar
Dm: deciduous molar
MIAME: Minimum Information About a Microarray Experiment
GEO: Gene Expression Omnibus
WGCNA: weighted correlation network analysis
PCA: principal component analysis
ANOVA: analysis of variance
qRT-PCR: real-time reverse transcription–polymerase chain reaction
LNA–DIG: locked nucleic acid–digoxigenin
