# Supplementary material for: Identification of differential microRNA expression during tooth morphogenesis in the heterodont dentition of miniature pigs, SusScrofa
Source: BMC Dev Biol. 2015 Dec 29;15:51. doi: 10.1186/s12861-015-0099-0 (PMC4696248; doi:10.1186/s12861-015-0099-0)
Supplement: Additional file 1: — Sample sets information in this study. (DOC 71 kb) [file 12861_2015_99_MOESM1_ESM.doc]

| **Additional file 1 Sample sets information in this study** | | | | | |
| --- | --- | --- | --- | --- | --- |
| **No.** | **Sample ID** | **Development stages** | **Time (day)** | **tooth types** | **Sample composition** |
| 1 | Di40 | Cap | 40 (after insemination) | the first deciduous incisor | mixture of 18 tooth germ tissues from three pregnant sows |
| 2 | Dc40 |  |  | deciduous canine | mixture of 18 tooth germ tissues from three pregnant sows |
| 3 | Dpm40 |  |  | the second deciduous premolar | mixture of 18 tooth germ tissues from three pregnant sows |
| 4 | Dm40 |  |  | deciduous molar | mixture of 18 tooth germ tissues from three pregnant sows |
| 5 | Di50 | Early bell | 50 (after insemination) | the first deciduous incisor | mixture of 15 tooth germ tissues from three pregnant sows |
| 6 | Dc50 |  |  | deciduous canine | mixture of 15 tooth germ tissues from three pregnant sows |
| 7 | Dpm50 |  |  | the second deciduous premolar | mixture of 15 tooth germ tissues from three pregnant sows |
| 8 | Dm50 |  |  | deciduous molar | mixture of 15 tooth germ tissues from three pregnant sows |
| 9 | Di60 | Late bell | 60 (after insemination) | the first deciduous incisor | mixture of 9 tooth germ tissues from three pregnant sows |
| 10 | Dc60 |  |  | deciduous canine | mixture of 9 tooth germ tissues from three pregnant sows |
| 11 | Dpm60 |  |  | the second deciduous premolar | mixture of 9 tooth germ tissues from three pregnant sows |
| 12 | Dm60 |  |  | deciduous molar | mixture of 9 tooth germ tissues from three pregnant sows |
