# Supplementary material for: Identification of differential microRNA expression during tooth morphogenesis in the heterodont dentition of miniature pigs, SusScrofa
Source: BMC Dev Biol. 2015 Dec 29;15:51. doi: 10.1186/s12861-015-0099-0 (PMC4696248; doi:10.1186/s12861-015-0099-0)
Supplement: Additional file 2: — Primer sequences of the real-time PCR experiments. (DOC 72 kb) [file 12861_2015_99_MOESM2_ESM.doc]

| **Additional file 2 Primer sequences of the real-time PCR experiments** | | | |
| --- | --- | --- | --- |
| **No.** | **miRNA Name** | **Primer** | **Primer Sequence** |
| 1 | ssc-miR-103 | SeqTence (5' to 3') | AGCAGCATTGTACAGGGCTATGA |
|  |  | RT primer | GTCGTATCCAGTGCAGGGTCCGAGGTATTCGCACTGGATACGACTCATAG |
|  |  | FW Primer | GCCGCCAGCAGCATTGTA |
|  |  | RV Primer | CGCAGGGTCCGAGGTATTC |
| 2 | ssc-miR-107 | SeqTence (5' to 3') | AGCAGCATTGTACAGGGCTATCA |
|  |  | RT primer | GTCGTATCCAGTGCAGGGTCCGAGGTATTCGCACTGGATACGACTGATAG |
|  |  | FW Primer | GCCGCCAGCAGCATTGTA |
|  |  | RV Primer | CGCAGGGTCCGAGGTATTC |
| 3 | ssc-miR-127 | SeqTence (5' to 3') | TCGGATCCGTCTGAGCTTGGCT |
|  |  | RT primer | CTATACCATAAGCGAGCAGTAGCGCGATGGTATAGAGCCAAGC |
|  |  | FW Primer | GTCCGTCGGATCCGTCTGAG |
|  |  | RV Primer | GCCATAAGCGAGCAGTAGCG |
| 4 | ssc-miR-133a | SeqTence (5' to 3') | TTGGTCCCCTTCAACCAGCTG |
|  |  | RT primer | GTCGTATCCAGTGCAGGGTCCGAGGTATTCGCACTGGATACGACCAGCTG |
|  |  | FW Primer | TTGCGTTGGTCCCCTTCA |
|  |  | RV Primer | CAGTGCAGGGTCCGAGGTAT |
| 5 | ssc-miR-133b | SeqTence (5' to 3') | TTTGGTCCCCTTCAACCAGCTAT |
|  |  | RT primer | CTATACCATAAGCGAGCAGTAGCGCGATGGTATAGATAGCTGG |
|  |  | FW Primer | GCGGCATTTGGTCCCCT |
|  |  | RV Primer | GCCATAAGCGAGCAGTAGCG |
| Control | ssc-miR-24 | SeqTence (5' to 3') | TGGCTCAGTTCAGCAGGAACAG |
|  |  | RT primer | GTCGTATCCAGTGCAGGGTCCGAGGTATTCGCACTGGATACGACCTGTTC |
|  |  | FW Primer | GCGGTGTTGGCTCAGTTCAG |
|  |  | RV Primer | CAGTGCAGGGTCCGAGGTATT |
