# Supplementary material for: Identification of differential microRNA expression during tooth morphogenesis in the heterodont dentition of miniature pigs, SusScrofa
Source: BMC Dev Biol. 2015 Dec 29;15:51. doi: 10.1186/s12861-015-0099-0 (PMC4696248; doi:10.1186/s12861-015-0099-0)
Supplement: Additional file 3: — LNA Probe sequences of five miRNAs. (DOC 30 kb) [file 12861_2015_99_MOESM3_ESM.doc]

| Probe Name | Sequence |
| --- | --- |
| ssc-mir-103,5`-DIG and 3`-DIG labeled | /5DigN/TCATAGCCCTGTACAATGCTGCT/3DigN/ |
| ssc-mir-107,5`-DIG and 3`-DIG labeled | /5DigN/TGATAGCCCTGTACAATGCTGCT/3DigN/ |
| ssc-mir-127,5`-DIG and 3`-DIG labeled | /5DigN/AGCCAAGCTCAGACGGATCCGA/3DigN/ |
| ssc-mir-133a,5`-DIG and 3`-DIG labeled | /5DigN/CAGCTGGTTGAAGGGGACCAA/3DigN/ |
| ssc-mir-133b,5`-DIG and 3`-DIG labeled | /5DigN/ATAGCTGGTTGAAGGGGACCAAA/3DigN/ |

**Additional file 3 Probe sequence used for in situ hybridization**
