# Supplementary material for: Identification of differential microRNA expression during tooth morphogenesis in the heterodont dentition of miniature pigs, SusScrofa
Source: BMC Dev Biol. 2015 Dec 29;15:51. doi: 10.1186/s12861-015-0099-0 (PMC4696248; doi:10.1186/s12861-015-0099-0)
Supplement: Additional file 4: — Detectable transcripts in different type tooth germ during different development stages. (DOC 1429 kb) [file 12861_2015_99_MOESM4_ESM.doc]

**Additional file 4 Detectable transcripts in different type tooth germ during different development stages**

| **No.** | **Probe_ID** | **Di40(260)** | **Di50(295)** | **Di60(281)** | **Dc40(370)** | **Dc50(427)** | **c60(304)** | **Dpm40(293)** | **Dpm50(270)** | **Dpm60(291)** | **Dm40(408)** | **Dm50(511)** | **Dm60(451)** |
| --- | --- | --- | --- | --- | --- | --- | --- | --- | --- | --- | --- | --- | --- |
| 1 | miR-100-3p-24550 |  |  |  |  |  |  |  |  |  |  | 36.83 |  |
| 2 | miR-101-3p-203 |  |  |  | 52.30 | 32.57 |  |  |  |  |  |  |  |
| 3 | **miR-103-5p-33548** | 20.25 | 72.52 | 61.39 | 78.03 | 41.94 | 102.14 | 22.17 | 47.35 | 73.77 | 91.11 | 34.62 |  |
| 4 | miR-105-3p-20193 |  |  |  | 47.57 |  |  |  |  |  |  | 30.52 |  |
| 5 | miR-105-5p-32318 |  |  |  |  | 18.22 |  |  |  |  |  | 26.64 |  |
| 6 | miR-105-5p-39093 |  |  |  |  |  |  |  |  |  |  |  |  |
| 7 | **miR-106a-5p-29928** | 247.90 | 408.17 | 506.60 | 447.75 | 1,136.48 | 208.04 | 133.12 | 312.78 | 318.22 | 308.21 | 1,405.21 | 1,007.25 |
| 8 | **miR-107-3p-33** | 1,224.20 | 1,968.19 | 2,397.70 | 2,192.01 | 1,860.74 | 1,770.12 | 959.27 | 1,843.28 | 1,721.01 | 1,663.25 | 2,611.03 | 2,776.14 |
| 9 | miR-10a-5p-40349 |  |  |  |  |  |  |  |  |  |  | 39.05 | 40.04 |
| 10 | miR-122-5p-1298 |  |  |  |  |  |  |  |  |  |  |  | 30.78 |
| 11 | miR-124-3p-1392 |  | 38.66 |  |  |  |  |  |  |  |  | 66.64 |  |
| 12 | miR-125a-3p-38755 |  |  |  | 43.12 | 17.51 |  |  |  |  | 36.31 | 55.24 | 42.72 |
| 13 | miR-125b-3p-8757 | 20.54 |  |  | 49.01 | 28.81 |  |  |  |  | 31.99 | 110.13 | 67.75 |
| 14 | **miR-126-3p-3530** | 216.46 | 794.60 | 469.37 | 589.88 | 1,134.90 | 546.39 | 1,155.98 | 195.58 | 477.09 | 346.91 | 1,301.32 | 1,888.26 |
| 15 | miR-126-5p-1328 |  |  |  |  | 16.18 |  |  |  |  |  | 60.59 | 78.02 |
| 16 | miR-127-5p-12072 |  |  |  |  |  |  |  |  |  |  |  |  |
| 17 | miR-128-5p-55018 |  |  |  |  | 17.14 |  |  |  |  |  | 41.81 |  |
| 18 | miR-129-5p-16749 |  |  |  | 36.22 |  |  |  |  |  | 29.09 | 46.92 |  |
| 19 | miR-1298-5p-26710 |  |  |  |  |  |  |  |  |  |  |  |  |
| 20 | miR-1306-3p-16142 |  |  |  |  | 21.82 |  |  |  |  |  | 98.54 | 96.08 |
| 21 | **miR-1307-3p-821** | 78.66 | 92.08 | 113.39 | 159.70 | 129.97 | 84.97 | 31.32 | 65.64 | 81.94 | 140.89 | 230.99 | 163.24 |
| 22 | miR-1307-5p-10307 |  |  |  | 43.12 |  |  |  |  |  | 45.61 | 66.38 | 51.23 |
| 23 | miR-1308-3p-49420 | 308.92 | 148.03 | 313.48 | 170.05 | 2,282.23 | 191.62 | 130.79 | 51.81 | 82.88 |  | 221.19 | 298.77 |
| 24 | miR-130a-5p-10408 |  |  |  | 29.45 | 23.26 | 28.64 |  |  |  |  | 41.94 | 51.31 |
| 25 | miR-130b-5p-1805 |  |  |  | 44.15 | 30.30 |  |  |  |  | 35.11 | 480.76 | 432.77 |
| 26 | miR-132-3p-16295 |  |  |  |  |  |  |  |  |  |  | 63.49 | 51.77 |
| 27 | miR-132-5p-38352 |  |  |  |  |  |  |  |  |  |  |  |  |
| 28 | **miR-133a-3p-1466** | 34.51 | 49.12 | 64.18 | 52.76 | 265.06 | 36.47 | 577.20 | 651.19 | 696.14 | 33.37 | 41.39 | 73.57 |
| 29 | miR-133b-3p-56920 |  | 74.41 | 65.32 | 44.24 | 239.35 | 42.55 | 356.43 | 652.78 | 557.97 | 21.86 |  | 41.60 |
| 30 | miR-135a-3p-15122 |  |  |  |  |  |  |  |  |  |  | 28.45 |  |
| 31 | miR-135a-3p-8771 |  |  |  | 29.43 |  |  |  |  |  |  | 39.20 | 23.98 |
| 32 | miR-135b-3p-42132 |  |  |  |  | 15.35 |  |  |  |  |  |  |  |
| 33 | miR-135b-5p-2350 |  | 63.37 | 39.61 | 54.18 | 27.93 |  |  |  |  | 27.79 |  |  |
| 34 | miR-136-3p-5100 |  |  |  |  |  |  |  |  |  |  |  |  |
| 35 | miR-138-3p-3259 |  | 39.91 | 48.84 | 32.76 | 47.66 |  |  | 48.31 |  |  | 130.45 | 137.79 |
| 36 | **miR-138-5p-826** | 221.68 | 553.01 | 405.45 | 248.04 | 299.44 | 209.42 | 50.93 | 396.41 | 225.12 | 191.21 | 137.95 | 116.96 |
| 37 | miR-139-3p-9531 |  |  |  |  |  |  |  |  |  |  | 44.08 | 53.08 |
| 38 | miR-139-5p-5165 |  |  | 40.71 | 30.22 | 27.99 |  |  |  |  | 76.27 | 657.89 | 1,068.79 |
| 39 | miR-1-3p-9331 |  |  |  |  |  |  |  |  |  |  |  |  |
| 40 | miR-142-5p-54139 |  |  |  |  |  |  |  |  |  |  |  |  |
| 41 | miR-143-5p-4039 |  |  |  |  |  |  |  |  |  |  |  | 36.25 |
| 42 | miR-144-3p-40226 |  | 23.77 |  |  |  |  |  |  |  |  |  |  |
| 43 | miR-144-5p-13256 |  |  |  |  |  |  |  |  |  |  |  |  |
| 44 | miR-145-3p-11588 |  |  |  |  |  |  |  |  |  |  |  |  |
| 45 | **miR-145-5p-234** | 391.06 | 1,605.71 | 1,027.44 | 650.79 | 743.71 | 1,369.94 | 462.69 | 1,226.40 | 1,623.51 | 1,072.83 | 1,383.98 | 1,237.54 |
| 46 | miR-146a-5p-5923 |  |  |  |  | 22.09 |  |  |  |  |  | 138.10 | 136.08 |
| 47 | miR-148a-5p-12537 |  |  | 45.65 | 45.96 |  | 56.01 |  |  | 41.18 | 76.95 |  |  |
| 48 | miR-148b-3p-24917 |  | 62.33 | 51.11 | 53.31 | 59.36 | 80.22 |  | 51.21 | 93.48 | 67.32 | 358.81 | 506.95 |
| 49 | **miR-149-3p-13400** | 6,722.92 | 3,279.72 | 3,585.84 | 10,765.03 | 6,094.70 | 4,375.00 | 4,582.84 | 4,160.21 | 2,486.36 | 10,967.34 | 8,448.01 | 7,086.04 |
| 50 | **miR-149-5p-11097** | 276.85 | 546.22 | 546.92 | 674.10 | 598.39 | 414.38 | 296.03 | 381.71 | 562.59 | 929.96 | 1,014.58 | 1,169.68 |
| 51 | miR-155-5p-11894 |  |  |  | 30.10 | 22.03 |  |  |  |  | 30.96 | 476.85 | 496.45 |
| 52 | **miR-16-5p-29943** | 11,236.76 | 13,040.69 | 12,952.16 | 15,541.13 | 1,430.43 | 15,720.85 | 10,066.29 | 17,670.23 | 22,934.07 | 24,212.39 | 3,047.85 | 3,696.22 |
| 53 | miR-17-3p-46722 |  |  |  | 36.49 | 16.46 |  |  |  |  | 74.28 | 47.57 | 49.65 |
| 54 | miR-181a-3p-3133 |  | 83.45 | 111.68 | 153.60 | 83.56 | 500.44 | 18.99 |  | 107.42 | 55.07 | 37.58 | 50.19 |
| 55 | **miR-181a-5p-39048** | 36.51 | 154.69 | 534.22 | 218.31 | 1,710.00 | 928.28 | 112.15 | 72.38 | 366.52 | 138.14 | 840.43 | 1,418.00 |
| 56 | **miR-181a-5p-39722** | 51.45 | 289.86 | 817.59 | 352.29 | 2,554.98 | 1,610.94 | 157.99 | 127.67 | 507.78 | 211.94 | 1,544.03 | 2,528.86 |
| 57 | miR-181b-3p-24826 |  |  |  |  |  |  |  |  |  |  | 39.02 |  |
| 58 | **miR-181b-5p-24202** | 142.15 | 310.01 | 806.72 | 401.88 | 1,005.69 | 882.30 | 191.68 | 210.11 | 543.35 | 369.46 | 2,685.10 | 3,418.07 |
| 59 | miR-181b-5p-34723 |  |  |  | 35.47 |  |  |  |  |  |  | 52.15 | 50.78 |
| 60 | miR-181c-3p-6759 |  | 44.14 |  | 45.03 | 23.85 |  |  |  |  | 43.03 | 35.18 | 38.47 |
| 61 | **miR-181c-5p-1684** | 476.15 | 1,079.70 | 1,533.30 | 1,676.47 | 2,858.18 | 2,604.34 | 964.39 | 534.01 | 1,195.73 | 1,566.26 | 2,017.62 | 3,305.41 |
| 62 | **miR-181d-5p-4330** | 159.72 | 283.74 | 703.85 | 428.11 | 692.81 | 795.69 | 190.14 | 217.86 | 506.97 | 437.11 | 2,223.59 | 2,791.76 |
| 63 | miR-182-3p-38717 |  |  |  |  |  |  |  |  |  |  |  |  |
| 64 | **miR-182-5p-1627** | 86.43 | 163.93 | 156.72 | 140.51 | 198.22 | 83.48 | 73.36 | 60.34 | 204.09 | 171.91 | 4,631.70 | 3,054.99 |
| 65 | miR-183-5p-749 | 22.19 | 44.92 | 45.60 | 31.99 | 37.70 |  |  |  | 49.87 | 53.94 | 1,440.72 | 1,000.08 |
| 66 | miR-187-3p-54578 |  |  | 41.19 | 44.11 | 43.84 |  |  |  |  | 30.33 | 42.43 | 61.91 |
| 67 | miR-187-5p-29087 |  |  |  | 42.02 | 22.24 |  |  |  |  | 38.80 | 53.63 |  |
| 68 | miR-191-3p-33349 |  |  |  |  |  |  |  |  |  |  | 37.77 |  |
| 69 | **miR-195-5p-6765** | 490.66 | 120.36 | 229.32 | 538.77 | 24.70 | 740.40 | 255.76 | 441.42 | 612.93 | 426.60 | 132.16 | 161.87 |
| 70 | miR-1983-3p-46851 |  | 37.65 |  | 38.20 | 24.45 | 76.16 | 28.29 | 55.05 |  |  | 36.51 |  |
| 71 | **miR-199a-3p-56318** | 631.04 | 1,857.98 | 1,767.00 | 940.58 | 1,842.75 | 894.19 | 639.46 | 1,107.03 | 1,982.25 | 1,002.27 | 10,997.68 | 12,169.52 |
| 72 | **miR-199b-5p-484** | 369.26 | 979.13 | 753.39 | 617.39 | 1,755.41 | 596.76 | 347.42 | 811.69 | 717.16 | 478.42 | 1,172.85 | 1,309.06 |
| 73 | miR-204-3p-7373 |  |  |  | 32.66 |  |  |  |  |  |  | 59.77 | 46.22 |
| 74 | miR-205-3p-2188 |  |  |  |  | 27.60 |  |  |  |  |  | 50.22 | 53.99 |
| 75 | **miR-205-5p-304** | 2,651.21 | 3,291.99 | 6,648.30 | 3,190.18 | 7,470.55 | 3,804.88 | 2,058.10 | 4,838.98 | 4,674.40 | 4,367.34 | 8,647.15 | 12,643.61 |
| 76 | miR-206-3p-33411 |  |  |  |  |  |  |  |  |  |  | 41.43 | 47.43 |
| 77 | **miR-20b-5p-17250** | 125.63 | 122.72 | 153.26 | 267.52 | 521.81 | 53.43 | 66.03 | 92.16 | 104.25 | 176.66 | 532.70 | 346.55 |
| 78 | miR-210-5p-32608 |  |  | 52.68 | 57.80 | 32.74 | 60.12 | 29.77 | 47.64 | 58.83 | 68.71 | 108.93 | 90.08 |
| 79 | miR-212-3p-41300 |  |  |  | 40.75 | 18.88 |  |  |  |  |  | 40.14 |  |
| 80 | miR-212-5p-17375 |  |  |  |  | 17.77 |  |  |  |  |  |  | 44.50 |
| 81 | miR-21-3p-58304 |  |  |  | 28.13 |  |  |  |  |  |  | 35.89 |  |
| 82 | **miR-2140-5p-46938** | 1,159.02 | 970.67 | 1,143.34 | 2,304.23 | 740.46 | 1,301.68 | 1,109.43 | 1,413.19 | 1,414.34 | 2,886.19 | 1,280.61 | 1,201.13 |
| 83 | **miR-214-3p-314** | 3,431.08 | 5,037.54 | 7,362.30 | 5,043.58 | 11,024.88 | 3,130.61 | 5,159.09 | 6,717.13 | 6,406.82 | 4,955.00 | 18,398.78 | 12,433.17 |
| 84 | **miR-214-5p-15474** | 125.39 | 146.61 | 166.17 | 379.58 | 25.77 | 204.57 | 110.73 | 551.38 | 793.69 | 1,310.93 | 50.33 | 36.65 |
| 85 | miR-215-5p-37456 |  |  |  |  |  |  |  |  |  |  |  | 40.89 |
| 86 | **miR-21-5p-44** | 70.74 | 244.24 | 253.37 | 111.17 | 190.00 | 148.33 | 92.20 | 98.46 | 174.30 | 83.81 | 2,850.06 | 2,935.18 |
| 87 | miR-216a-5p-54378 |  |  |  |  |  |  |  |  |  |  |  |  |
| 88 | miR-216b-5p-9660 |  |  |  |  | 17.42 |  |  |  |  |  |  |  |
| 89 | miR-217-5p-12828 |  |  |  |  |  |  |  |  |  |  |  |  |
| 90 | miR-218-3p-20231 |  | 27.16 |  |  | 20.48 |  |  |  |  |  |  |  |
| 91 | miR-218-5p-18206 | 222.67 |  | 214.79 | 367.70 | 139.22 |  |  | 169.04 |  |  | 425.79 |  |
| 92 | miR-218-5p-53526 |  |  | 46.08 | 41.33 | 26.91 |  | 17.90 |  | 49.62 | 49.58 | 699.43 | 741.30 |
| 93 | miR-219-3p-56939 |  |  |  |  |  |  |  |  |  |  |  |  |
| 94 | **miR-221-3p-121** | 299.87 | 569.39 | 754.27 | 658.03 | 437.32 | 657.26 | 199.21 | 574.14 | 722.60 | 1,002.13 | 1,014.62 | 879.40 |
| 95 | miR-221-5p-3246 |  |  |  |  |  |  |  |  |  |  |  |  |
| 96 | miR-2320-3p-50422 |  |  |  |  | 20.71 |  |  |  |  |  | 53.32 | 50.08 |
| 97 | **miR-2320-5p-8854** | 52.68 | 117.93 | 123.90 | 157.53 | 64.11 | 184.09 | 45.49 | 113.91 | 159.86 | 311.88 | 103.00 | 98.25 |
| 98 | miR-2366-3p-29027 |  |  |  |  | 17.89 |  |  |  |  |  | 42.23 |  |
| 99 | **miR-23a-3p-217** | 398.27 | 699.68 | 1,138.03 | 652.40 | 734.96 | 453.64 | 269.55 | 498.07 | 868.75 | 626.52 | 9,453.38 | 11,638.27 |
| 100 | miR-23a-5p-7460 |  |  |  |  | 18.39 |  |  |  |  |  | 40.44 | 54.56 |
| 101 | miR-23b-5p-427 |  |  |  |  |  |  |  |  |  |  | 106.59 | 78.12 |
| 102 | miR-2411-3p-35411 |  |  |  | 41.17 | 28.15 |  |  |  |  |  |  |  |
| 103 | miR-24-5p-12205 |  |  |  |  |  |  |  |  |  |  | 31.13 |  |
| 104 | miR-24-5p-54188 |  |  |  |  |  |  |  |  |  |  | 30.53 |  |
| 105 | miR-2483-3p-8160 |  |  |  | 26.94 |  |  |  |  |  |  | 37.77 | 50.79 |
| 106 | **miR-27a-3p-1818** | 157.74 | 209.27 | 235.66 | 249.43 | 178.75 | 117.16 | 99.87 | 115.56 | 164.42 | 195.33 | 2,107.12 | 2,456.33 |
| 107 | miR-27a-5p-18747 |  |  |  |  |  |  |  |  |  |  |  |  |
| 108 | miR-28-5p-30328 | 33.86 | 47.96 | 51.36 | 41.40 | 85.51 |  |  |  | 41.91 | 48.80 | 521.23 | 615.18 |
| 109 | **miR-2887-5p-29252** | 675.96 | 432.05 | 440.10 | 1,472.29 | 411.68 | 606.81 | 604.18 | 504.66 | 455.45 | 1,324.15 | 1,163.64 | 1,151.49 |
| 110 | **miR-2904-5p-20636** | 2,623.28 | 1,863.65 | 1,595.58 | 3,359.50 | 2,379.59 | 1,738.24 | 2,265.93 | 1,098.59 | 1,152.34 | 1,917.96 | 25,567.22 | 24,793.71 |
| 111 | **miR-296-3p-176** | 61.92 | 72.64 | 108.40 | 253.62 | 64.94 | 132.88 | 48.73 | 142.35 | 192.18 | 504.33 | 167.60 | 129.57 |
| 112 | miR-29a-3p-669 |  |  |  |  |  |  |  |  |  |  |  | 39.13 |
| 113 | miR-29b-3p-19778 |  | 28.58 |  | 43.27 | 20.58 |  |  |  |  | 23.81 |  |  |
| 114 | miR-29c-5p-34060 |  |  |  | 41.07 | 15.99 |  |  |  |  |  |  |  |
| 115 | miR-301b-3p-21966 | 29.96 | 55.66 |  | 97.38 | 19.40 | 36.84 | 19.31 | 61.28 | 67.76 | 126.61 |  |  |
| 116 | miR-301b-5p-25757 |  |  |  |  | 17.56 |  |  |  |  |  |  |  |
| 117 | miR-30a-5p-186 | 45.52 | 150.64 | 112.65 | 94.45 | 171.53 | 67.55 | 94.12 | 79.39 | 133.38 | 88.36 | 480.33 | 618.46 |
| 118 | miR-30b-3p-22935 |  |  |  | 33.96 |  |  |  |  |  |  | 30.74 | 37.07 |
| 119 | miR-30c-3p-5525 |  |  |  | 32.79 |  |  |  |  |  |  | 47.63 |  |
| 120 | miR-30c-3p-9651 |  | 28.89 |  |  |  |  |  |  |  |  |  |  |
| 121 | **miR-30c-5p-2750** | 182.77 | 373.55 | 489.39 | 276.59 | 439.19 | 296.11 | 141.49 | 276.50 | 474.63 | 363.90 | 3,075.33 | 3,194.07 |
| 122 | **miR-31-5p-42472** | 183.99 | 140.87 | 143.98 | 613.16 | 336.99 | 94.67 | 284.03 | 83.80 | 257.07 | 282.65 | 970.54 | 428.29 |
| 123 | **miR-320-3p-5** | 622.51 | 671.01 | 1,473.47 | 1,401.06 | 1,145.29 | 856.25 | 374.58 | 1,358.90 | 1,316.58 | 2,108.74 | 4,322.53 | 3,207.17 |
| 124 | miR-324-3p-38854 |  |  |  | 38.55 | 19.90 |  |  |  |  | 69.26 | 86.07 | 89.43 |
| 125 | miR-325-3p-52014 |  |  |  | 34.51 |  |  |  |  |  |  | 30.64 | 54.78 |
| 126 | miR-326-3p-20734 | 20.04 |  | 64.55 | 60.47 |  | 65.04 | 30.15 | 90.58 | 89.68 | 208.82 | 61.01 | 114.29 |
| 127 | miR-335-3p-18172 |  |  |  | 48.80 |  |  |  |  |  |  | 36.17 |  |
| 128 | miR-339-3p-22035 |  |  |  |  | 16.55 |  |  |  |  |  | 31.14 | 53.68 |
| 129 | **miR-339-5p-24465** | 253.73 | 570.29 | 502.12 | 581.29 | 148.79 | 812.86 | 419.51 | 832.09 | 1,127.33 | 1,509.85 | 135.68 | 198.53 |
| 130 | **miR-342-3p-3575** | 38.26 | 128.84 | 122.73 | 78.94 | 98.40 | 102.68 | 32.28 | 90.27 | 146.39 | 135.86 | 871.68 | 899.54 |
| 131 | miR-342-5p-4956 |  |  |  | 44.97 | 25.92 |  |  |  |  | 48.50 | 101.15 | 58.63 |
| 132 | miR-345-5p-26266 |  |  |  | 101.97 |  | 58.91 | 32.65 | 50.16 | 180.79 | 414.08 |  |  |
| 133 | miR-34b-3p-37237 |  |  |  |  | 18.31 |  |  |  |  |  | 28.77 | 49.41 |
| 134 | miR-34b-5p-14844 |  | 32.79 |  | 41.81 | 21.13 |  |  |  |  | 42.99 |  |  |
| 135 | miR-34c-3p-39896 |  |  |  | 36.73 | 19.83 |  |  |  |  |  | 54.18 | 46.18 |
| 136 | miR-363-3p-495 | 20.85 | 51.64 | 46.23 | 43.97 | 74.07 | 64.27 |  |  | 59.05 | 41.14 | 99.68 | 102.06 |
| 137 | miR-363-5p-23718 |  |  |  |  |  |  |  |  |  |  |  | 34.25 |
| 138 | miR-365-5p-11544 |  |  |  | 58.53 |  |  |  |  |  | 47.44 | 39.09 |  |
| 139 | miR-365-5p-8303 |  |  |  |  |  |  |  |  |  |  |  |  |
| 140 | miR-370-3p-2334 |  |  |  | 52.83 | 30.28 |  | 36.37 |  | 38.82 | 46.87 | 77.10 | 46.39 |
| 141 | miR-370-5p-25384 |  |  |  |  | 23.54 |  |  |  |  |  |  |  |
| 142 | miR-374b-3p-16579 |  |  |  |  |  |  |  |  |  |  |  |  |
| 143 | miR-378-3p-21764 |  |  | 42.46 | 44.07 | 42.27 |  | 21.23 | 48.28 |  |  | 36.91 |  |
| 144 | miR-384-3p-4651 |  |  |  |  | 19.22 |  |  |  |  |  |  |  |
| 145 | miR-421-3p-5678 |  |  |  |  | 27.38 |  |  |  |  |  | 121.82 | 123.98 |
| 146 | miR-424-5p-2477 |  |  |  |  |  |  |  |  |  |  |  |  |
| 147 | miR-429-3p-734 | 28.06 |  | 65.97 | 48.18 | 74.95 |  | 26.30 |  | 92.04 | 61.63 | 380.44 | 509.19 |
| 148 | miR-429-5p-55061 |  | 33.63 |  |  | 20.07 |  |  |  |  |  | 34.88 | 46.43 |
| 149 | miR-431-3p-38023 | 22.51 |  |  | 36.80 | 27.47 |  |  |  |  |  | 42.36 | 44.23 |
| 150 | miR-431-5p-35690 |  | 28.07 |  |  | 50.48 |  | 40.09 |  | 46.03 |  |  |  |
| 151 | miR-432-5p-807 |  |  |  | 37.31 | 34.79 |  |  |  |  | 18.35 | 273.62 | 128.46 |
| 152 | miR-449a-5p-16490 |  |  |  |  |  |  |  |  |  |  |  |  |
| 153 | **miR-451-5p-792** | 257.73 | 3,581.55 | 345.85 | 521.10 | 1,952.11 | 1,045.26 | 970.71 | 297.52 | 403.76 | 106.12 | 683.72 | 545.08 |
| 154 | miR-452-3p-48902 |  |  |  |  |  |  |  |  |  |  |  |  |
| 155 | miR-452-5p-863 |  |  |  |  | 19.35 |  |  |  |  |  |  |  |
| 156 | miR-454-3p-9708 |  |  |  |  | 22.27 |  |  |  |  |  | 96.67 | 114.62 |
| 157 | miR-455-5p-4494 |  | 57.01 |  | 39.99 | 46.24 |  |  |  |  | 32.38 |  |  |
| 158 | miR-488-5p-33542 |  |  |  |  | 16.45 |  |  |  |  |  |  |  |
| 159 | miR-490-3p-8253 |  |  |  |  |  |  |  |  |  |  | 36.94 |  |
| 160 | miR-491-5p-13995 |  | 36.35 | 58.33 | 71.87 | 16.91 | 63.06 | 20.77 | 75.36 | 86.27 | 292.35 | 38.33 |  |
| 161 | miR-493-5p-3333 |  |  |  |  |  |  |  |  |  |  | 82.71 | 71.89 |
| 162 | miR-497-5p-5546 | 51.86 | 148.44 | 123.77 | 110.95 | 46.55 | 132.18 | 51.97 | 103.38 | 146.11 | 119.53 | 44.60 |  |
| 163 | miR-503-3p-26240 |  |  |  |  |  |  |  |  |  | 20.18 |  |  |
| 164 | miR-504-3p-35231 | 28.13 |  |  | 61.18 |  |  |  | 46.38 |  | 58.04 | 53.63 | 54.16 |
| 165 | miR-504-5p-623 |  |  | 45.94 | 95.44 |  | 55.74 | 96.17 | 76.17 | 267.67 | 498.15 | 76.28 | 64.37 |
| 166 | miR-505-3p-3920 |  | 42.31 | 58.83 | 51.59 | 54.46 |  |  | 55.60 | 86.06 | 79.60 | 552.72 | 481.71 |
| 167 | miR-505-5p-1305 |  |  |  |  |  |  |  |  |  |  | 128.12 | 72.19 |
| 168 | miR-551a-3p-4326 | 96.65 | 152.51 | 197.67 | 255.58 | 29.17 | 233.86 | 50.49 | 255.85 | 372.26 | 721.13 | 44.85 | 45.48 |
| 169 | miR-551a-5p-37472 |  |  |  |  | 21.45 |  |  |  |  | 29.68 |  |  |
| 170 | miR-582-5p-57143 |  | 28.97 |  |  | 19.49 |  |  |  |  |  | 32.45 |  |
| 171 | miR-671-5p-23875 |  |  |  |  |  |  |  |  |  |  | 38.09 | 40.41 |
| 172 | miR-676-5p-36205 |  |  |  |  |  |  |  |  |  |  |  |  |
| 173 | miR-708-3p-18309 |  |  |  |  |  |  |  |  |  |  |  |  |
| 174 | miR-708-5p-10346 |  |  |  |  |  |  |  |  |  |  |  |  |
| 175 | miR-7-3p-45112 |  |  |  |  | 20.62 |  |  |  |  |  |  |  |
| 176 | miR-769-5p-11763 |  |  |  | 40.55 |  |  |  |  |  | 28.29 | 36.15 |  |
| 177 | miR-7a-3p-22829 |  |  |  | 30.14 | 18.67 |  |  |  |  |  |  |  |
| 178 | **miR-7a-5p-430** | 131.41 | 275.13 | 532.03 | 245.07 | 409.73 | 275.58 | 96.79 | 204.94 | 528.86 | 408.29 | 13,054.51 | 16,673.24 |
| 179 | **miR-7d-3p-4142** | 43.15 | 84.69 | 85.32 | 57.96 | 76.46 | 68.42 | 33.21 | 76.14 | 78.56 | 117.90 | 612.67 | 571.88 |
| 180 | **miR-7d-5p-35** | 89.28 | 180.06 | 361.87 | 183.37 | 306.47 | 206.85 | 60.49 | 157.88 | 356.92 | 338.82 | 11,838.17 | 12,872.89 |
| 181 | miR-7e-3p-28921 |  |  |  |  |  |  |  |  |  |  | 31.53 | 38.98 |
| 182 | **miR-7f-5p-692** | 55.40 | 114.60 | 228.84 | 111.58 | 190.28 | 123.75 | 38.25 | 91.02 | 269.56 | 202.55 | 11,088.95 | 13,745.44 |
| 183 | **miR-7i-5p-8** | 460.47 | 950.47 | 1,461.03 | 672.89 | 1,211.27 | 676.39 | 455.07 | 758.48 | 1,508.39 | 967.95 | 11,468.55 | 13,062.09 |
| 184 | miR-873-3p-49454 |  |  |  |  |  |  |  |  |  |  |  |  |
| 185 | miR-874-3p-24775 |  |  |  | 49.53 | 24.56 |  |  |  |  | 73.09 | 89.05 | 87.40 |
| 186 | **miR-92a-3p-56231** | 45.85 | 83.49 | 125.80 | 98.02 | 302.17 | 70.44 | 50.54 | 68.64 | 183.87 | 146.23 | 6,657.14 | 4,189.53 |
| 187 | miR-92a-5p-27412 |  |  |  |  |  |  |  |  |  |  |  |  |
| 188 | miR-92a-5p-52622 |  |  |  |  | 22.81 |  |  |  |  |  | 48.04 | 49.12 |
| 189 | miR-92b-5p-11484 |  |  |  |  |  |  |  |  |  |  | 51.47 |  |
| 190 | **miR-96-5p-13194** | 40.78 | 171.29 | 77.53 | 92.18 | 144.59 | 63.10 | 214.59 | 65.86 | 114.22 | 94.18 | 55.36 | 59.75 |
| 191 | miR-98-3p-22500 |  |  |  |  |  |  |  |  |  |  |  |  |
| 192 | miR-99a-3p-2459 | 26.68 |  | 56.45 | 79.91 |  | 55.22 | 25.54 | 75.58 | 158.60 | 341.35 | 32.41 |  |
| 193 | miR-99b-3p-543 |  |  | 41.23 | 34.14 | 30.86 |  |  |  |  | 46.17 | 51.48 | 45.34 |
| 194 | **PC-103-3p-49493** | 1,423.37 | 2,374.21 | 2,742.19 | 2,434.90 | 2,145.95 | 2,150.59 | 1,050.39 | 2,199.63 | 2,031.11 | 1,886.36 | 2,815.45 | 2,952.81 |
| 195 | **PC-103-5p-40816** | 874.66 | 916.09 | 1,226.84 | 1,468.95 | 1,390.08 | 881.18 | 536.50 | 818.91 | 740.28 | 1,009.70 | 1,905.91 | 2,106.48 |
| 196 | PC-125b-5p-29588 |  | 34.48 |  | 38.47 | 22.43 | 52.52 | 19.06 | 48.49 |  | 59.05 | 58.77 | 62.30 |
| 197 | **PC-138-5p-9675** | 163.28 | 439.79 | 284.93 | 143.28 | 244.37 | 167.62 | 31.30 | 334.18 | 155.20 | 127.90 | 112.00 | 86.66 |
| 198 | **PC-15a-5p-46278** | 361.81 | 245.80 | 229.84 | 870.80 | 48.14 | 497.47 | 122.67 | 688.77 | 827.38 | 839.34 | 182.36 | 235.73 |
| 199 | PC-22-3p-57394 |  | 48.33 | 55.77 | 41.18 | 34.46 | 149.23 |  | 55.72 | 47.42 | 32.38 | 50.77 | 113.23 |
| 200 | PC-27b-3p-40686 |  |  |  |  |  |  |  |  |  |  |  |  |
| 201 | **PC-296-3p-9208** | 192.81 | 170.86 | 185.45 | 692.92 | 141.83 | 238.51 | 131.29 | 266.60 | 332.26 | 1,353.40 | 523.22 | 395.77 |
| 202 | PC-326-3p-33634 |  |  |  |  |  |  |  |  |  |  | 33.22 |  |
| 203 | PC-345-3p-22893 |  |  |  | 43.36 | 28.89 |  | 15.22 |  |  | 55.63 | 41.79 | 46.07 |
| 204 | PC-371-5p-22929 | 48.67 |  |  | 106.22 | 26.01 |  | 64.96 |  |  | 96.72 | 36.48 | 38.56 |
| 205 | PC-3p-12333 |  |  |  |  |  |  |  |  |  |  | 29.61 |  |
| 206 | PC-3p-15885 |  |  |  |  |  |  |  |  |  |  |  |  |
| 207 | **PC-3p-174** | 26.68 | 49.00 | 67.39 | 46.72 | 73.01 | 65.09 | 34.77 | 53.10 | 75.53 | 97.83 | 572.29 | 619.39 |
| 208 | PC-3p-18428 |  |  |  |  |  |  |  |  |  |  |  |  |
| 209 | PC-3p-19139 |  |  |  |  |  |  |  |  |  |  |  | 48.95 |
| 210 | **PC-3p-19163** | 2,509.58 | 2,635.90 | 2,489.84 | 4,934.52 | 2,039.49 | 2,742.43 | 3,039.60 | 2,870.03 | 2,960.13 | 6,994.90 | 1,437.81 | 1,726.00 |
| 211 | **PC-3p-25529** | 3,267.05 | 1,744.61 | 2,812.70 | 7,777.75 | 2,897.50 | 2,318.99 | 1,970.61 | 2,549.33 | 2,104.29 | 5,176.23 | 1,834.40 | 1,372.40 |
| 212 | PC-3p-2582 |  |  |  | 32.11 |  |  |  |  |  | 32.80 | 47.13 | 56.60 |
| 213 | PC-3p-26099 |  |  |  | 53.58 |  |  |  |  |  | 71.02 | 77.08 | 69.91 |
| 214 | PC-3p-26672 |  |  |  |  |  |  |  |  |  |  |  |  |
| 215 | **PC-3p-27055** | 15,773.80 | 9,489.62 | 13,266.77 | 22,338.27 | 12,887.27 | 14,204.33 | 12,368.80 | 19,918.66 | 11,483.10 | 28,889.81 | 11,740.40 | 10,611.14 |
| 216 | PC-3p-28725 |  |  |  |  |  |  |  |  |  |  |  | 65.01 |
| 217 | PC-3p-28844 |  |  |  |  |  |  |  |  |  |  |  |  |
| 218 | PC-3p-29296 |  |  |  | 28.08 | 22.18 |  |  |  |  |  | 121.07 | 62.57 |
| 219 | PC-3p-29999 |  |  |  |  |  |  |  |  |  |  |  |  |
| 220 | **PC-3p-30694** | 1,182.26 | 508.57 | 246.65 | 3,425.07 | 854.53 | 319.57 | 913.45 | 237.61 | 207.81 | 2,617.02 | 733.90 | 614.01 |
| 221 | **PC-3p-30961** | 580.55 | 451.49 | 865.43 | 1,783.22 | 627.30 | 671.25 | 423.04 | 1,170.57 | 572.32 | 1,026.70 | 949.92 | 510.21 |
| 222 | PC-3p-31237 |  |  |  |  |  |  |  |  |  |  |  |  |
| 223 | PC-3p-32533 |  |  |  |  |  |  |  |  |  |  |  |  |
| 224 | **PC-3p-32678** | 278.30 | 218.39 | 277.29 | 660.50 | 192.99 | 475.47 | 407.41 | 201.24 | 317.08 | 761.17 | 230.24 | 266.89 |
| 225 | PC-3p-33106 | 50.35 | 36.12 |  | 150.64 |  | 63.43 | 60.85 |  |  | 262.45 | 247.47 | 261.02 |
| 226 | PC-3p-33750 | 31.89 |  |  | 48.45 | 17.89 |  | 32.09 |  |  | 59.33 | 81.42 | 65.15 |
| 227 | PC-3p-33990 |  |  |  |  |  |  |  |  |  |  | 41.14 | 43.06 |
| 228 | PC-3p-34178 |  |  |  |  |  |  |  |  |  |  |  |  |
| 229 | PC-3p-34812 |  |  |  |  |  |  |  |  |  |  | 30.41 |  |
| 230 | PC-3p-34905 |  |  |  |  |  |  |  |  |  |  |  |  |
| 231 | PC-3p-35116 |  |  |  |  |  |  |  |  |  |  |  |  |
| 232 | PC-3p-35409 |  |  |  |  |  |  |  |  |  |  |  |  |
| 233 | PC-3p-35429 |  |  |  | 53.39 |  |  |  |  |  | 89.29 | 60.62 | 38.10 |
| 234 | PC-3p-36665 |  |  |  |  |  |  |  |  |  |  |  |  |
| 235 | **PC-3p-36835** | 397.21 | 173.45 | 81.10 | 1,266.70 | 257.88 | 159.93 | 349.28 | 122.01 | 108.18 | 2,019.50 | 530.06 | 428.91 |
| 236 | PC-3p-37277 |  |  |  |  |  |  |  |  |  |  |  |  |
| 237 | PC-3p-37654 |  |  |  |  |  |  |  |  |  |  | 68.27 | 66.28 |
| 238 | PC-3p-38954 |  |  |  |  |  |  |  |  |  |  |  |  |
| 239 | **PC-3p-39857** | 18,489.56 | 15,263.92 | 13,980.18 | 26,650.42 | 12,437.30 | 17,320.85 | 20,146.95 | 19,328.64 | 14,170.48 | 23,435.31 | 7,756.68 | 7,799.68 |
| 240 | PC-3p-39976 |  |  |  |  |  |  |  |  |  |  |  |  |
| 241 | PC-3p-40135 |  |  |  |  |  |  |  |  |  |  |  |  |
| 242 | **PC-3p-40159** | 982.28 | 315.60 | 389.87 | 2,264.05 | 875.34 | 516.37 | 603.39 | 662.38 | 282.65 | 1,540.38 | 541.20 | 313.66 |
| 243 | PC-3p-40586 |  |  |  |  |  |  |  |  |  |  |  |  |
| 244 | PC-3p-40640 | 36.49 |  |  | 41.89 | 73.10 | 65.12 | 47.58 |  |  | 42.44 | 86.24 | 50.15 |
| 245 | PC-3p-40847 |  |  |  |  |  |  |  |  |  |  | 39.76 | 53.30 |
| 246 | PC-3p-40920 |  |  |  |  |  |  |  |  |  |  | 44.93 | 54.80 |
| 247 | PC-3p-41121 |  |  |  |  |  |  |  |  |  |  |  |  |
| 248 | **PC-3p-41498** | 154.19 | 59.48 | 64.58 | 259.83 | 64.55 | 61.35 | 129.41 | 54.48 | 57.45 | 707.16 | 5,941.69 | 5,660.03 |
| 249 | PC-3p-41956 |  |  |  |  |  |  |  |  |  |  |  |  |
| 250 | PC-3p-42894 |  |  |  |  |  |  |  |  |  |  | 41.55 | 55.87 |
| 251 | PC-3p-43104 |  |  |  |  |  |  |  |  |  |  |  |  |
| 252 | PC-3p-43512 |  |  |  |  |  |  |  |  |  |  |  |  |
| 253 | PC-3p-44175 |  |  |  |  |  |  |  |  |  |  |  |  |
| 254 | PC-3p-44897 |  |  |  |  |  |  |  |  |  |  |  |  |
| 255 | **PC-3p-45009** | 12,978.01 | 7,066.65 | 9,120.14 | 10,791.56 | 9,076.43 | 8,534.72 | 14,292.87 | 9,788.02 | 6,826.27 | 10,787.60 | 24,032.70 | 18,748.86 |
| 256 | PC-3p-45597 |  |  |  | 32.13 |  |  |  |  |  |  |  |  |
| 257 | PC-3p-46523 | 99.94 | 52.31 | 49.38 | 249.20 | 41.54 |  | 71.62 |  | 43.93 | 201.41 | 277.84 | 227.91 |
| 258 | PC-3p-46977 |  |  |  |  |  |  |  |  |  |  | 34.68 |  |
| 259 | PC-3p-48110 | 28.06 |  |  | 71.22 |  |  | 43.60 |  |  | 122.33 | 234.60 | 195.16 |
| 260 | PC-3p-48419 |  |  |  |  |  |  |  |  |  | 54.33 |  |  |
| 261 | **PC-3p-48957** | 429.87 | 103.08 | 65.53 | 1,133.59 | 162.84 | 62.10 | 330.57 | 49.23 | 56.62 | 1,681.73 | 226.15 | 201.79 |
| 262 | PC-3p-49234 |  |  |  |  |  |  | 11.80 |  |  | 41.62 | 49.28 |  |
| 263 | PC-3p-49285 | 68.10 |  |  | 114.54 | 28.43 | 38.55 | 50.02 |  |  | 165.17 | 189.85 | 181.13 |
| 264 | PC-3p-49387 |  |  |  | 29.25 |  |  |  |  |  |  | 48.57 | 46.25 |
| 265 | PC-3p-49488 |  |  |  |  |  |  |  |  |  |  |  |  |
| 266 | PC-3p-49615 |  |  |  |  |  |  |  |  |  |  |  |  |
| 267 | PC-3p-49747 |  |  |  |  |  |  |  |  |  |  |  |  |
| 268 | PC-3p-49776 |  |  |  |  |  |  |  |  |  |  |  |  |
| 269 | PC-3p-50740 |  |  |  |  |  |  |  |  |  |  |  |  |
| 270 | **PC-3p-51289** | 489.62 | 436.87 | 685.32 | 1,509.13 | 468.09 | 559.44 | 436.26 | 837.48 | 483.95 | 981.13 | 621.80 | 470.74 |
| 271 | PC-3p-51876 |  |  |  |  |  |  |  |  |  |  |  |  |
| 272 | PC-3p-51982 |  |  |  |  |  |  |  |  |  | 20.20 | 35.98 |  |
| 273 | PC-3p-53154 |  |  |  |  |  |  |  |  |  |  |  |  |
| 274 | PC-3p-53437 | 88.38 | 56.28 |  | 133.51 | 48.10 | 51.37 | 75.58 |  | 58.00 | 364.91 | 2,644.26 | 2,294.95 |
| 275 | PC-3p-53455 |  |  |  | 34.38 |  |  |  |  |  |  | 47.36 |  |
| 276 | PC-3p-53970 |  |  |  |  |  |  |  |  |  |  |  |  |
| 277 | PC-3p-54511 |  |  |  |  |  |  |  |  |  | 48.97 | 333.22 | 379.48 |
| 278 | PC-3p-55239 |  |  |  | 55.70 |  |  |  |  |  | 60.88 | 55.82 | 62.05 |
| 279 | PC-3p-5588 |  |  |  |  |  |  |  |  |  |  |  |  |
| 280 | PC-3p-56283 |  |  |  |  |  |  |  |  |  |  |  |  |
| 281 | **PC-3p-56303** | 238.66 | 124.43 | 74.70 | 812.56 | 144.72 | 141.39 | 216.71 | 135.68 | 62.02 | 805.03 | 871.13 | 646.29 |
| 282 | PC-3p-56416 |  |  |  |  |  |  |  |  |  |  |  |  |
| 283 | PC-3p-56435 |  |  |  |  |  |  |  |  |  |  |  |  |
| 284 | PC-3p-56517 |  |  |  | 52.77 |  |  |  |  |  | 117.14 | 63.70 | 39.04 |
| 285 | PC-3p-56768 |  |  |  |  |  |  |  |  |  |  |  |  |
| 286 | PC-3p-57569 |  |  |  |  | 29.10 |  |  |  |  |  | 46.19 |  |
| 287 | PC-3p-57648 |  |  |  |  |  |  |  |  |  |  |  |  |
| 288 | PC-3p-58149 |  |  |  | 29.97 |  |  |  |  |  |  | 104.58 | 68.85 |
| 289 | PC-3p-58208 |  |  |  | 56.16 |  |  |  |  |  | 50.74 | 339.85 | 385.90 |
| 290 | PC-3p-58626 |  |  |  |  |  |  |  |  |  |  |  |  |
| 291 | PC-3p-58735 |  |  |  |  |  | 27.84 |  |  |  | 58.16 | 46.85 | 52.95 |
| 292 | PC-3p-58746 |  |  |  |  |  |  |  |  |  | 20.44 |  | 33.37 |
| 293 | PC-3p-58754 |  |  |  |  |  |  |  |  |  |  |  |  |
| 294 | PC-3p-7119 |  |  |  | 30.03 |  |  |  |  |  |  |  | 38.99 |
| 295 | PC-3p-7694 |  |  |  |  |  |  |  |  |  |  | 36.23 | 54.38 |
| 296 | PC-3p-8038 |  |  |  |  |  |  |  |  |  |  |  |  |
| 297 | **PC-3p-8280** | 6,366.62 | 2,096.96 | 2,878.80 | 2,709.77 | 3,342.37 | 3,212.55 | 5,154.39 | 2,029.69 | 3,550.24 | 4,195.31 | 25,561.68 | 18,226.66 |
| 298 | **PC-5p-10248** | 2,932.18 | 1,580.92 | 687.71 | 7,212.66 | 1,948.64 | 734.28 | 2,031.13 | 624.32 | 996.53 | 7,223.23 | 2,124.10 | 1,342.79 |
| 299 | PC-5p-11488 |  |  |  |  |  |  |  |  |  |  |  |  |
| 300 | **PC-5p-16513** | 1,049.72 | 854.05 | 1,074.97 | 2,407.97 | 712.27 | 1,184.49 | 939.20 | 1,175.93 | 848.04 | 1,933.16 | 510.76 | 467.12 |
| 301 | PC-5p-17308 |  |  |  |  |  |  |  |  |  |  | 38.25 | 46.95 |
| 302 | PC-5p-2320 | 23.61 | 48.50 | 47.80 | 57.32 | 28.26 | 55.58 |  |  | 51.83 | 70.21 | 215.13 | 218.12 |
| 303 | PC-5p-24478 |  |  |  |  |  |  |  |  |  |  |  |  |
| 304 | PC-5p-24688 |  |  |  | 49.63 | 21.59 |  |  |  |  | 40.00 | 52.75 |  |
| 305 | PC-5p-24896 |  |  |  |  |  |  |  |  |  |  |  |  |
| 306 | **PC-5p-26373** | 10,414.37 | 9,720.04 | 8,333.92 | 9,881.04 | 8,263.28 | 8,536.66 | 8,961.84 | 10,889.79 | 10,286.72 | 20,340.93 | 15,056.97 | 6,045.12 |
| 307 | PC-5p-27556 |  |  |  | 36.24 |  |  |  |  |  |  |  |  |
| 308 | PC-5p-28165 |  |  |  |  |  |  |  |  |  |  | 35.34 |  |
| 309 | PC-5p-28606 |  |  |  |  |  |  |  |  |  |  | 44.14 | 38.29 |
| 310 | PC-5p-31989 |  |  |  |  |  |  |  |  |  |  |  |  |
| 311 | PC-5p-32141 |  |  |  |  |  |  |  |  |  |  | 38.20 | 46.96 |
| 312 | PC-5p-32379 | 47.40 | 41.24 |  | 128.31 | 24.03 | 55.10 | 57.73 |  |  | 226.23 | 278.25 | 277.83 |
| 313 | **PC-5p-32513** | 98.92 | 50.64 | 51.30 | 368.40 | 31.29 | 107.95 | 120.66 | 89.09 | 72.17 | 602.18 | 267.68 | 100.62 |
| 314 | PC-5p-33325 |  |  |  | 26.86 |  |  |  |  |  | 17.42 |  |  |
| 315 | PC-5p-33511 |  |  |  |  |  |  |  |  |  |  |  |  |
| 316 | PC-5p-33677 |  |  |  | 69.01 |  |  | 31.02 |  |  | 98.57 | 237.52 | 250.76 |
| 317 | PC-5p-33921 |  |  |  |  |  |  |  |  |  |  |  |  |
| 318 | PC-5p-34181 |  |  |  |  |  |  |  |  |  |  |  |  |
| 319 | PC-5p-35585 |  |  |  |  |  |  |  |  |  |  |  |  |
| 320 | PC-5p-36274 |  |  |  |  |  |  |  |  |  |  |  |  |
| 321 | PC-5p-36531 | 159.24 | 54.50 | 44.21 | 276.33 | 81.95 | 53.41 | 129.14 | 49.86 |  | 508.09 | 2,137.12 | 1,774.41 |
| 322 | PC-5p-37380 |  |  |  |  |  |  |  |  |  |  | 26.79 |  |
| 323 | PC-5p-38477 |  |  |  |  |  |  |  |  |  |  |  |  |
| 324 | PC-5p-38629 |  |  |  |  |  |  |  |  |  |  |  |  |
| 325 | PC-5p-38663 |  |  |  |  |  |  |  |  |  |  | 41.91 |  |
| 326 | PC-5p-39068 | 45.67 |  |  | 58.13 | 26.26 | 34.68 | 33.79 |  |  | 81.27 | 69.10 | 53.61 |
| 327 | PC-5p-39118 |  |  |  |  |  |  |  |  |  |  |  |  |
| 328 | PC-5p-41031 |  | 23.39 |  | 55.74 |  |  |  |  |  | 73.79 | 463.70 | 492.98 |
| 329 | **PC-5p-41107** | 575.17 | 828.58 | 693.67 | 1,166.81 | 1,885.20 | 573.99 | 650.48 | 330.62 | 411.08 | 666.50 | 2,222.07 | 588.81 |
| 330 | PC-5p-41213 |  |  |  |  |  |  |  |  |  |  | 33.94 |  |
| 331 | PC-5p-41788 |  |  |  |  |  |  |  |  |  |  |  |  |
| 332 | PC-5p-41806 |  |  |  |  |  |  |  |  |  |  |  |  |
| 333 | PC-5p-42342 |  |  |  |  |  |  |  |  |  |  | 97.76 |  |
| 334 | PC-5p-42602 |  |  |  |  |  |  |  |  |  |  | 85.21 | 680.10 |
| 335 | PC-5p-42817 |  |  |  |  |  |  |  |  |  |  | 40.29 |  |
| 336 | PC-5p-43067 |  |  |  | 30.05 |  |  |  |  |  |  | 26.15 |  |
| 337 | PC-5p-43364 |  |  |  | 34.88 |  |  |  |  |  | 28.53 |  |  |
| 338 | PC-5p-43899 | 302.59 | 61.11 |  | 811.60 | 112.81 | 70.90 | 212.54 |  | 46.52 | 734.02 | 369.81 | 329.81 |
| 339 | PC-5p-44178 |  |  |  |  |  |  |  |  |  |  |  |  |
| 340 | PC-5p-44199 |  |  |  |  |  |  |  |  |  |  |  |  |
| 341 | **PC-5p-44440** | 5,625.37 | 5,114.84 | 7,047.89 | 10,226.43 | 6,381.70 | 8,020.34 | 4,869.39 | 10,147.50 | 6,181.35 | 11,617.85 | 5,502.58 | 4,871.74 |
| 342 | PC-5p-44729 | 171.22 |  |  | 205.82 | 34.87 | 35.02 | 132.85 |  | 55.03 | 798.26 | 547.02 | 544.46 |
| 343 | PC-5p-44827 |  |  |  |  |  |  |  |  |  |  |  |  |
| 344 | PC-5p-45059 |  |  |  | 29.98 |  |  |  |  |  |  |  |  |
| 345 | PC-5p-45735 |  |  |  |  |  |  |  |  |  |  |  | 53.65 |
| 346 | PC-5p-45980 |  |  |  |  |  |  |  |  |  |  |  |  |
| 347 | PC-5p-46602 |  |  |  | 56.98 | 24.73 | 34.07 | 31.70 |  |  | 66.95 | 61.74 | 56.37 |
| 348 | **PC-5p-46964** | 1,508.68 | 2,023.48 | 1,679.58 | 3,443.42 | 1,012.72 | 2,246.34 | 1,452.94 | 2,331.28 | 1,417.24 | 3,411.08 | 1,288.37 | 1,343.26 |
| 349 | PC-5p-46978 |  |  |  |  |  |  |  |  |  |  |  |  |
| 350 | PC-5p-47542 |  |  |  |  |  |  |  |  |  |  |  |  |
| 351 | **PC-5p-47796** | 106.76 | 102.19 | 115.57 | 272.30 | 80.25 | 302.39 | 110.09 | 204.37 | 155.97 | 328.34 | 73.85 | 83.74 |
| 352 | PC-5p-48202 |  |  |  |  |  |  |  |  |  |  |  |  |
| 353 | PC-5p-48483 |  |  |  |  |  |  |  |  |  |  |  |  |
| 354 | **PC-5p-48616** | 345.80 | 68.92 | 70.81 | 1,144.34 | 299.25 | 170.36 | 216.46 | 126.06 | 69.89 | 702.86 | 238.34 | 123.05 |
| 355 | PC-5p-48686 |  |  |  |  |  |  |  |  |  |  |  |  |
| 356 | **PC-5p-48980** | 476.83 | 480.54 | 374.18 | 914.31 | 247.95 | 579.24 | 549.51 | 485.15 | 389.69 | 848.76 | 197.20 | 182.47 |
| 357 | PC-5p-49190 |  |  |  |  |  |  |  |  |  |  |  |  |
| 358 | **PC-5p-49283** | 280.34 | 90.91 | 54.62 | 631.74 | 110.10 | 56.67 | 195.65 | 57.10 | 50.39 | 912.83 | 3,695.46 | 3,434.47 |
| 359 | PC-5p-49827 |  |  |  |  |  |  |  |  |  | 27.62 | 62.04 | 41.77 |
| 360 | PC-5p-51030 |  |  |  |  |  |  |  |  |  |  |  |  |
| 361 | PC-5p-51130 |  |  |  |  |  |  |  |  |  |  |  |  |
| 362 | PC-5p-51164 |  |  |  |  | 16.09 |  |  |  |  |  |  |  |
| 363 | PC-5p-51903 |  |  |  |  | 19.69 |  |  |  |  | 23.27 |  |  |
| 364 | PC-5p-52238 |  |  |  | 51.42 |  |  |  |  |  | 33.39 | 43.76 | 42.94 |
| 365 | **PC-5p-52539** | 1,106.48 | 897.34 | 1,067.51 | 2,437.50 | 719.74 | 1,131.40 | 987.30 | 1,084.97 | 901.57 | 1,957.39 | 507.81 | 457.98 |
| 366 | PC-5p-52592 |  |  |  |  |  |  |  |  |  |  |  |  |
| 367 | PC-5p-53374 |  |  |  | 41.49 |  |  |  |  |  | 58.69 | 37.51 | 62.22 |
| 368 | PC-5p-53415 |  |  |  | 30.35 |  |  |  |  |  |  | 190.92 | 226.00 |
| 369 | PC-5p-53739 |  |  |  | 40.14 |  |  |  |  |  | 21.36 | 37.16 |  |
| 370 | PC-5p-53953 | 54.96 |  |  | 142.41 | 31.89 | 61.05 | 44.96 | 44.77 |  | 270.94 | 1,038.08 | 1,014.94 |
| 371 | **PC-5p-54435** | 512.89 | 346.17 | 230.19 | 1,467.21 | 375.20 | 785.45 | 532.08 | 607.97 | 264.37 | 1,544.10 | 1,030.91 | 731.33 |
| 372 | PC-5p-55150 |  |  |  |  |  |  |  |  |  |  |  |  |
| 373 | **PC-5p-55329** | 700.89 | 348.90 | 226.12 | 1,462.86 | 516.08 | 343.21 | 537.14 | 661.73 | 231.54 | 810.75 | 443.60 | 287.89 |
| 374 | PC-5p-55358 |  |  |  |  |  |  |  |  |  |  |  |  |
| 375 | PC-5p-55708 |  |  |  |  |  |  |  |  |  |  |  |  |
| 376 | PC-5p-56327 |  |  |  |  |  |  |  |  |  |  |  |  |
| 377 | PC-5p-56505 |  |  |  |  |  |  |  |  |  |  |  |  |
| 378 | PC-5p-57824 |  |  |  |  |  |  |  |  |  |  |  |  |
| 379 | **PC-5p-57957** | 1,353.37 | 500.48 | 424.55 | 3,078.51 | 892.02 | 613.76 | 985.43 | 640.92 | 325.38 | 3,248.57 | 1,208.41 | 933.88 |
| 380 | PC-5p-57993 |  |  |  |  |  |  |  |  |  |  | 32.41 |  |
| 381 | **PC-5p-58250** | 927.92 | 758.00 | 628.67 | 1,364.04 | 870.13 | 650.84 | 636.17 | 424.44 | 366.55 | 786.24 | 783.44 | 907.65 |
| 382 | PC-5p-58510 | 29.46 |  |  | 106.43 |  | 34.47 | 20.03 |  |  | 108.85 | 63.15 | 64.06 |
| 383 | PC-5p-58593 |  |  |  |  |  |  |  |  |  |  |  |  |
| 384 | PC-5p-58681 | 58.14 | 36.19 |  | 148.31 | 19.77 |  | 69.98 |  |  | 615.73 | 136.30 | 141.73 |
| 385 | PC-5p-58697 |  |  |  |  |  |  |  |  |  |  |  |  |
| 386 | PC-5p-58824 |  |  |  |  |  |  |  |  |  |  |  |  |
| 387 | PC-5p-6028 | 41.63 |  |  | 101.10 | 26.56 |  |  |  |  | 61.85 | 77.73 | 64.24 |
| 388 | PC-5p-6438 |  |  |  |  |  |  |  |  |  |  |  |  |
| 389 | PC-5p-7882 |  |  |  |  |  |  |  |  |  |  |  |  |
| 390 | PC-5p-8848 |  |  |  |  |  |  |  |  |  |  |  |  |
| 391 | PC-5p-9054 |  |  |  |  |  |  |  |  |  |  |  |  |
| 392 | PC-5p-9764 |  |  |  |  |  |  |  |  |  |  |  |  |
| 393 | **PC-7b-5p-10** | 51.98 | 144.09 | 229.64 | 101.62 | 219.05 | 140.11 | 53.04 | 102.70 | 255.96 | 217.66 | 9,402.45 | 9,762.30 |
| 394 | PC-7c-5p-34268 |  |  |  |  |  |  |  |  |  |  | 773.64 | 515.00 |
| 395 | PC-7e-5p-58372 |  | 72.82 | 118.73 | 41.22 | 120.05 | 72.55 |  | 63.39 | 104.49 | 81.90 | 3,162.92 | 2,279.05 |
| 396 | PC-7f-3p-54949 |  |  |  |  |  |  |  |  |  |  | 3,250.61 | 2,775.09 |
| 397 | **PC-7g-5p-3653** | 53.85 | 116.67 | 175.41 | 67.67 | 251.76 | 115.45 | 35.35 | 73.14 | 232.33 | 154.28 | 7,680.51 | 8,557.05 |
| 398 | **PN-100-5p-58610** | 73.53 | 221.27 | 255.93 | 132.07 | 335.68 | 146.18 | 112.09 | 89.40 | 268.54 | 179.29 | 2,362.01 | 2,446.60 |
| 399 | **PN-103-3p-30235** | 891.56 | 1,258.19 | 1,795.68 | 1,474.20 | 1,496.36 | 1,026.14 | 568.95 | 1,092.62 | 1,038.22 | 929.07 | 1,927.64 | 2,047.06 |
| 400 | **PN-106b-3p-1913** | 72.91 | 147.61 | 128.97 | 243.94 | 92.56 | 166.34 | 47.82 | 103.47 | 209.60 | 516.03 | 222.73 | 222.96 |
| 401 | **PN-106b-5p-439** | 137.64 | 362.43 | 220.47 | 279.34 | 458.76 | 138.97 | 128.81 | 214.08 | 228.05 | 196.06 | 535.59 | 491.94 |
| 402 | PN-1193-5p-10059 |  |  |  |  |  |  |  |  |  |  | 43.73 |  |
| 403 | PN-1197-3p-3514 |  |  |  |  |  |  | 15.95 |  |  |  |  |  |
| 404 | PN-1224-5p-3082 |  |  |  | 47.71 | 25.21 | 69.45 | 30.92 |  |  | 123.12 | 103.83 | 128.65 |
| 405 | PN-1224-5p-34251 |  | 36.29 |  | 43.01 | 27.04 | 60.97 | 29.87 |  | 69.71 | 112.47 | 94.44 | 109.76 |
| 406 | PN-1247-3p-11661 |  |  |  |  |  |  |  |  |  |  | 49.37 | 46.54 |
| 407 | **PN-1247-5p-2591** | 99.88 | 116.77 | 182.93 | 127.44 | 142.54 | 139.95 | 84.28 | 164.86 | 193.70 | 218.10 | 454.52 | 503.09 |
| 408 | PN-1261-5p-12486 | 31.16 |  |  | 44.96 |  | 42.23 | 28.71 |  |  |  | 43.63 |  |
| 409 | PN-1261-5p-3406 |  |  |  | 61.77 | 21.54 | 43.36 | 25.22 |  |  |  | 31.08 | 41.12 |
| 410 | **PN-1271-5p-24855** | 22.33 | 57.95 | 58.93 | 41.81 | 62.83 | 66.38 | 21.18 | 54.29 | 79.65 | 84.44 | 852.33 | 634.18 |
| 411 | **PN-1274a-5p-54245** | 538.09 | 1,338.18 | 602.60 | 1,453.54 | 414.25 | 1,470.23 | 839.37 | 1,114.37 | 565.61 | 482.65 | 134.48 | 95.28 |
| 412 | **PN-1274b-5p-24235** | 6,161.25 | 7,914.48 | 3,250.94 | 11,638.34 | 5,577.99 | 4,115.79 | 7,515.02 | 3,219.34 | 2,733.23 | 4,527.87 | 1,427.97 | 1,078.16 |
| 413 | **PN-1274b-5p-339** | 3,120.83 | 5,849.91 | 2,851.55 | 5,938.23 | 2,923.02 | 4,873.50 | 4,530.62 | 4,176.11 | 2,682.76 | 2,623.95 | 890.76 | 776.51 |
| 414 | PN-1357-5p-50913 |  | 45.71 |  | 29.68 | 45.20 | 64.02 | 21.74 |  | 43.20 | 44.14 | 123.42 | 78.70 |
| 415 | **PN-1385-3p-36413** | 437.64 | 366.53 | 427.04 | 1,190.01 | 329.86 | 684.67 | 326.09 | 621.87 | 322.32 | 916.71 | 212.92 | 173.24 |
| 416 | PN-138b-3p-30491 |  |  |  |  |  |  |  |  |  |  | 27.35 |  |
| 417 | PN-1-3p-13186 |  | 30.53 |  |  | 68.79 |  | 47.87 |  | 169.88 |  | 222.71 | 267.83 |
| 418 | **PN-140-3p-18** | 552.96 | 1,159.39 | 1,910.74 | 1,385.52 | 75.99 | 1,673.93 | 520.43 | 884.40 | 1,539.50 | 3,221.44 | 101.36 | 196.54 |
| 419 | PN-140-5p-18632 |  |  |  |  | 16.58 |  |  |  |  |  |  |  |
| 420 | PN-140-5p-5323 |  | 48.47 |  | 27.57 | 48.40 | 46.68 |  |  |  |  | 28.31 | 46.61 |
| 421 | PN-141-3p-21462 | 158.48 | 178.63 | 140.83 | 542.59 | 35.52 | 448.11 | 139.31 | 109.36 | 384.86 | 162.55 |  |  |
| 422 | **PN-151-5p-5539** | 73.38 | 223.93 | 248.70 | 124.68 | 228.98 | 180.61 | 116.38 | 109.75 | 275.00 | 188.49 | 1,783.01 | 1,857.37 |
| 423 | **PN-15a-5p-24695** | 1,288.08 | 1,323.22 | 1,317.07 | 2,353.48 | 130.67 | 1,961.90 | 849.91 | 2,655.87 | 2,972.67 | 3,028.18 | 302.39 | 435.04 |
| 424 | **PN-181b-5p-4329** | 139.78 | 284.25 | 662.84 | 362.42 | 675.74 | 775.42 | 154.30 | 186.69 | 449.30 | 356.00 | 1,975.39 | 2,687.77 |
| 425 | **PN-181c-5p-27101** | 139.27 | 186.39 | 595.89 | 497.82 | 643.71 | 593.33 | 175.36 | 138.79 | 395.85 | 411.59 | 1,958.24 | 2,548.14 |
| 426 | **PN-1826-3p-701** | 25,381.87 | 19,777.79 | 26,156.06 | 25,194.65 | 20,310.16 | 31,216.43 | 29,043.67 | 29,486.57 | 31,833.48 | 45,683.60 | 19,034.08 | 17,505.27 |
| 427 | PN-1827-3p-10407 |  |  | 49.94 | 40.36 | 45.85 | 35.14 |  |  | 65.96 | 51.34 | 7,419.19 | 7,843.05 |
| 428 | PN-1842-3p-46163 |  |  |  |  |  |  |  |  |  |  | 34.00 |  |
| 429 | **PN-1842-5p-9566** | 42.01 | 133.80 | 100.37 | 88.89 | 48.67 | 95.24 | 37.92 | 70.47 | 155.72 | 150.99 | 131.80 | 104.50 |
| 430 | PN-185-5p-50418 |  | 113.31 | 95.99 | 70.03 | 77.31 | 155.02 | 45.42 | 67.94 | 95.34 | 76.00 | 255.95 | 258.29 |
| 431 | PN-188-5p-10334 |  | 41.50 |  | 51.21 | 20.19 | 50.12 | 24.14 |  |  | 74.13 | 125.39 | 142.23 |
| 432 | **PN-1937b-5p-17039** | 25,378.68 | 28,841.49 | 19,656.35 | 29,817.24 | 17,366.68 | 29,806.68 | 30,011.84 | 27,709.10 | 13,943.38 | 11,211.19 | 10,729.34 | 8,028.19 |
| 433 | **PN-193b-3p-948** | 327.34 | 880.50 | 981.95 | 1,329.61 | 210.61 | 1,480.62 | 574.26 | 807.70 | 1,428.39 | 1,667.36 | 398.86 | 498.92 |
| 434 | PN-193b-5p-4411 |  |  |  | 42.94 |  |  |  |  |  | 31.81 | 51.32 | 51.02 |
| 435 | **PN-195-5p-30300** | 3,431.28 | 2,533.45 | 2,712.42 | 5,250.47 | 92.82 | 5,578.99 | 2,258.42 | 3,780.22 | 6,416.45 | 5,404.33 | 378.45 | 392.47 |
| 436 | PN-1959-5p-58783 |  |  | 53.52 | 55.41 | 94.24 | 59.35 | 26.53 |  |  | 50.08 | 235.30 | 136.98 |
| 437 | **PN-199c-3p-271** | 461.19 | 738.10 | 756.56 | 695.25 | 936.88 | 282.85 | 469.56 | 341.19 | 929.97 | 717.98 | 10,554.94 | 11,166.55 |
| 438 | **PN-200a-3p-145** | 1,770.20 | 2,750.99 | 2,474.83 | 4,893.91 | 450.26 | 3,899.84 | 1,994.83 | 1,772.21 | 5,055.75 | 3,560.21 | 431.65 | 791.55 |
| 439 | PN-200a-5p-7019 |  |  |  | 29.15 | 18.58 |  |  |  |  |  | 36.96 | 54.58 |
| 440 | **PN-200b-3p-110** | 46.18 | 68.44 | 109.22 | 83.07 | 131.37 | 78.85 | 27.23 | 59.61 | 155.79 | 125.78 | 3,127.19 | 5,058.05 |
| 441 | **PN-200b-3p-161** | 42.30 | 75.89 | 138.83 | 85.13 | 133.78 | 114.01 | 29.77 | 50.92 | 170.34 | 136.88 | 3,050.12 | 5,044.46 |
| 442 | PN-200b-5p-2366 |  |  |  | 27.97 | 23.62 |  |  |  |  |  | 193.85 | 271.99 |
| 443 | **PN-200c-3p-193** | 47.37 | 45.99 | 102.55 | 59.97 | 96.37 | 120.26 | 21.40 | 50.56 | 148.13 | 108.44 | 3,163.76 | 4,449.77 |
| 444 | **PN-203-3p-308** | 291.00 | 48.38 | 62.18 | 100.13 | 64.69 | 44.56 | 16.05 | 190.15 | 40.26 | 42.12 | 926.40 | 1,158.41 |
| 445 | **PN-205-5p-5764** | 2,288.73 | 3,080.65 | 5,983.85 | 2,811.52 | 6,547.33 | 3,860.25 | 1,822.21 | 4,615.90 | 4,204.67 | 3,823.56 | 7,850.64 | 11,616.91 |
| 446 | **PN-2134-3p-3545** | 626.51 | 404.56 | 256.04 | 515.41 | 452.20 | 444.14 | 653.09 | 197.78 | 220.24 | 449.29 | 25,804.11 | 20,738.66 |
| 447 | **PN-2138-5p-36627** | 2,651.21 | 2,133.03 | 2,373.48 | 4,412.98 | 2,805.54 | 2,794.02 | 2,383.20 | 2,319.00 | 2,037.69 | 3,949.48 | 11,269.88 | 11,705.31 |
| 448 | PN-223-3p-48223 |  |  |  |  |  |  |  |  |  | 28.79 |  |  |
| 449 | **PN-2478-3p-31772** | 7,050.09 | 12,048.11 | 7,799.94 | 15,262.60 | 9,393.65 | 13,379.45 | 10,089.86 | 12,769.90 | 6,224.05 | 10,708.92 | 12,355.34 | 9,411.04 |
| 450 | PN-25-3p-13660 |  |  |  | 31.65 | 47.08 |  |  |  |  | 23.50 | 1,385.63 | 1,021.61 |
| 451 | **PN-25-3p-43** | 89.66 | 251.61 | 396.52 | 174.37 | 410.87 | 176.76 | 60.38 | 161.24 | 353.96 | 231.93 | 6,295.24 | 3,864.12 |
| 452 | PN-25-5p-584 |  | 37.32 |  | 51.13 | 19.86 | 48.48 |  |  |  | 57.31 | 38.40 |  |
| 453 | **PN-26b-5p-197** | 40.70 | 51.62 | 74.64 | 53.19 | 102.01 | 66.32 | 34.51 | 60.27 | 86.10 | 106.70 | 7,779.90 | 11,208.12 |
| 454 | PN-299-3p-365 |  |  |  | 29.66 | 22.53 |  |  |  |  |  | 38.78 |  |
| 455 | **PN-299-5p-13746** | 23.39 | 65.19 | 77.40 | 41.66 | 89.84 | 57.69 | 48.57 | 99.06 | 127.39 | 46.47 | 346.98 | 293.46 |
| 456 | **PN-300-3p-13997** | 393.74 | 212.55 | 230.78 | 931.42 | 35.24 | 248.97 | 1,048.93 | 2,029.80 | 2,096.80 | 2,180.25 | 139.14 | 150.11 |
| 457 | PN-301-5p-2668 |  |  |  |  | 24.48 | 38.72 |  |  |  |  | 33.91 | 29.90 |
| 458 | PN-301a-3p-20657 | 126.53 | 501.76 | 160.93 | 234.02 | 400.55 | 233.69 | 159.99 | 144.47 | 98.58 | 76.87 |  |  |
| 459 | PN-3168-5p-14157 |  |  |  |  |  |  |  |  |  |  |  |  |
| 460 | **PN-320b-3p-5073** | 632.91 | 712.87 | 1,512.02 | 1,392.84 | 1,204.33 | 901.96 | 406.22 | 1,384.54 | 1,359.30 | 2,221.34 | 4,323.53 | 3,540.19 |
| 461 | **PN-320c-3p-11241** | 577.17 | 771.90 | 1,538.07 | 1,393.22 | 1,207.33 | 820.06 | 384.89 | 1,346.65 | 1,296.93 | 2,142.21 | 4,269.12 | 3,409.40 |
| 462 | **PN-320c-3p-599** | 574.04 | 722.89 | 1,549.79 | 1,398.89 | 1,175.36 | 883.62 | 370.79 | 1,309.21 | 1,254.84 | 2,246.13 | 4,244.72 | 3,359.73 |
| 463 | **PN-320d-3p-2200** | 441.62 | 530.22 | 1,099.51 | 1,034.22 | 874.72 | 646.75 | 276.04 | 967.71 | 1,000.67 | 1,419.20 | 3,187.75 | 2,414.96 |
| 464 | PN-323-5p-6736 |  |  |  |  |  |  |  |  |  |  | 32.68 | 55.38 |
| 465 | PN-323b-3p-1142 |  |  |  |  | 22.21 |  |  |  |  |  | 52.84 | 46.84 |
| 466 | PN-323b-5p-58192 |  |  |  |  |  |  |  |  |  |  |  |  |
| 467 | PN-32-5p-39476 |  |  |  |  | 16.91 |  |  |  |  | 20.86 |  |  |
| 468 | PN-329-5p-10165 |  |  |  |  | 20.67 |  |  |  |  | 43.21 |  |  |
| 469 | PN-329-5p-11345 |  |  |  |  |  |  |  | 43.70 |  | 87.64 |  |  |
| 470 | PN-329a-3p-1371 |  | 38.84 |  | 44.73 | 39.91 | 40.83 |  | 56.00 | 83.14 | 34.66 | 214.44 | 189.68 |
| 471 | PN-329a-3p-32943 |  |  |  |  | 23.79 |  |  |  |  |  | 55.67 | 55.33 |
| 472 | **PN-330-3p-177** | 29.64 | 56.00 | 56.62 | 89.00 | 24.83 | 92.72 | 32.17 | 74.43 | 83.65 | 168.27 | 84.10 | 86.51 |
| 473 | PN-330-3p-2129 |  | 44.50 | 70.28 | 93.28 | 20.57 | 85.23 | 26.77 |  | 78.82 | 164.37 | 90.28 | 87.70 |
| 474 | PN-33-3p-4671 |  |  |  |  | 22.72 |  |  |  |  |  | 29.06 | 35.97 |
| 475 | PN-33b-5p-1238 |  |  |  | 42.74 | 17.46 |  |  |  | 35.99 | 49.80 |  |  |
| 476 | PN-33b-5p-55245 |  |  |  |  |  |  |  |  |  |  |  |  |
| 477 | **PN-3596-5p-30871** | 42.55 | 78.91 | 186.39 | 103.02 | 161.51 | 116.79 | 36.45 | 67.18 | 217.24 | 212.19 | 11,188.10 | 11,964.21 |
| 478 | PN-362-3p-16716 |  |  |  |  | 26.42 |  |  |  |  |  | 37.15 |  |
| 479 | PN-362-5p-2229 |  |  |  |  | 35.48 | 37.44 |  | 56.14 |  |  | 32.29 | 56.76 |
| 480 | PN-369-3p-33810 |  |  |  |  |  |  |  |  |  |  |  |  |
| 481 | PN-369-5p-3770 |  |  |  |  | 21.34 |  |  |  |  |  | 39.06 |  |
| 482 | PN-375-3p-3473 |  |  |  | 38.21 | 24.32 |  |  |  |  |  | 185.63 | 51.07 |
| 483 | PN-376b-3p-13138 |  |  |  |  | 24.01 |  | 130.50 |  |  |  |  |  |
| 484 | PN-376c-3p-2496 |  | 92.60 | 70.22 | 79.96 | 110.94 | 60.19 | 319.37 | 119.12 | 144.64 | 58.54 | 133.70 | 112.84 |
| 485 | PN-376c-5p-43489 |  |  |  |  |  |  |  |  |  |  |  |  |
| 486 | PN-376d-3p-9586 |  |  |  | 27.58 | 20.30 |  | 51.93 |  |  |  | 34.40 |  |
| 487 | PN-377-3p-18696 |  |  |  |  |  |  |  |  |  |  |  | 146.22 |
| 488 | PN-377-5p-6676 |  |  |  | 40.38 |  |  | 21.10 | 49.05 | 59.56 | 78.05 | 38.86 |  |
| 489 | PN-378-5p-25042 |  | 38.54 | 39.96 | 48.15 | 49.73 | 49.09 | 31.64 |  | 37.70 | 29.08 | 45.48 | 44.23 |
| 490 | PN-379-3p-17668 |  | 31.73 |  |  | 31.51 | 33.33 |  |  | 33.32 |  | 199.79 | 78.62 |
| 491 | PN-379-5p-1421 |  |  |  |  | 41.37 |  |  |  | 51.77 | 32.37 | 957.49 | 441.09 |
| 492 | PN-380-3p-267 |  |  |  |  | 20.52 | 27.07 |  |  |  |  | 408.00 | 248.94 |
| 493 | PN-380-5p-1636 |  |  |  |  | 18.98 |  |  |  |  |  | 36.82 |  |
| 494 | **PN-381-3p-1991** | 416.07 | 286.84 | 285.04 | 948.77 | 28.07 | 304.00 | 1,096.47 | 2,577.51 | 2,391.48 | 2,188.71 | 155.46 | 154.84 |
| 495 | **PN-381-5p-15017** | 28.82 | 72.18 | 79.56 | 46.96 | 51.27 | 86.60 | 34.50 | 130.28 | 109.05 | 58.65 | 60.19 | 47.95 |
| 496 | PN-382-3p-1007 |  |  |  |  |  |  |  |  |  |  | 88.12 | 78.74 |
| 497 | PN-382-5p-139 |  |  |  |  | 41.40 |  |  | 44.99 | 58.54 |  | 757.86 | 492.19 |
| 498 | PN-383-5p-1411 |  |  |  |  |  |  |  |  |  |  |  |  |
| 499 | PN-409-3p-335 |  | 60.92 | 110.21 | 32.45 | 72.85 | 47.00 | 45.09 | 65.65 | 91.81 | 70.00 | 824.92 | 360.87 |
| 500 | PN-409-3p-5527 |  | 46.62 | 65.40 | 52.87 | 59.67 | 48.47 | 39.41 | 74.31 | 78.64 | 49.58 | 844.89 | 379.11 |
| 501 | PN-409-5p-873 |  |  |  |  | 23.84 |  |  |  |  |  | 49.08 | 48.69 |
| 502 | PN-410-3p-4677 |  |  |  |  |  |  |  |  |  |  |  |  |
| 503 | PN-410-5p-17791 |  |  |  |  |  |  |  |  |  |  |  |  |
| 504 | PN-411-3p-3887 | 21.82 |  | 50.75 | 42.27 | 30.66 |  |  |  | 37.85 |  | 209.21 | 67.24 |
| 505 | **PN-411-5p-771** | 164.30 | 457.64 | 162.02 | 298.26 | 190.45 | 162.27 | 629.76 | 281.15 | 191.67 | 78.50 | 40.15 | 32.32 |
| 506 | PN-412-5p-4020 |  | 25.32 |  |  | 21.36 |  |  |  |  |  | 55.02 | 48.27 |
| 507 | PN-429-3p-16007 |  | 48.89 | 74.85 | 38.64 | 67.63 | 69.42 |  | 54.83 | 69.49 | 49.50 | 350.44 | 469.55 |
| 508 | **PN-429-3p-6578** | 30.53 | 63.60 | 84.27 | 58.32 | 75.49 | 62.65 | 23.19 |  | 85.18 | 61.35 | 411.92 | 563.74 |
| 509 | PN-484-5p-2677 | 34.27 |  | 66.16 | 36.99 | 36.02 | 84.92 | 26.20 | 82.75 | 43.54 | 78.45 | 123.50 | 139.83 |
| 510 | **PN-485-3p-375** | 88.37 | 152.39 | 124.12 | 124.24 | 142.47 | 112.71 | 253.24 | 377.28 | 262.14 | 172.82 | 269.89 | 167.90 |
| 511 | PN-485-5p-400 |  |  |  |  | 23.59 |  |  |  |  |  | 58.41 | 36.49 |
| 512 | PN-486-5p-52346 |  |  |  |  | 18.71 |  |  |  |  |  | 46.15 | 41.27 |
| 513 | PN-487b-3p-443 | 26.56 | 46.17 |  | 52.28 | 79.36 | 49.10 | 51.72 | 72.11 | 95.31 | 56.24 | 445.38 | 318.38 |
| 514 | PN-487b-5p-28138 |  |  |  |  |  |  |  |  |  |  | 35.09 |  |
| 515 | **PN-494-3p-3917** | 1,366.61 | 542.44 | 213.35 | 2,778.90 | 412.71 | 317.39 | 1,505.87 | 106.46 | 234.37 | 335.78 | 5,528.91 | 7,005.33 |
| 516 | PN-494-5p-15099 |  |  |  |  |  |  |  |  |  |  |  |  |
| 517 | **PN-495-3p-530** | 27.34 | 56.49 | 57.89 | 33.54 | 98.87 | 44.48 | 64.91 | 55.23 | 131.42 | 47.13 | 424.38 | 318.85 |
| 518 | PN-496-3p-58900 |  |  |  |  | 21.20 |  |  |  | 53.87 | 28.75 |  |  |
| 519 | PN-500-3p-3905 |  |  |  |  | 18.20 | 37.93 |  |  |  |  | 49.29 | 58.11 |
| 520 | **PN-500-5p-8209** | 46.13 | 88.06 | 103.57 | 91.71 | 79.73 | 121.64 | 74.29 | 106.42 | 137.80 | 146.79 | 98.94 | 92.71 |
| 521 | PN-501-3p-39962 |  |  |  |  | 17.39 |  |  |  |  |  | 44.85 | 67.86 |
| 522 | PN-509-5p-29637 |  |  |  |  |  |  |  |  |  |  |  |  |
| 523 | PN-532-5p-23788 |  |  |  |  | 21.28 |  |  |  |  |  | 46.11 | 55.24 |
| 524 | PN-539-3p-9544 |  | 63.90 |  |  |  | 45.82 |  |  | 59.71 |  |  | 72.46 |
| 525 | PN-539-5p-31092 |  |  |  |  | 25.74 |  |  |  |  |  | 99.31 | 67.21 |
| 526 | PN-541-3p-44064 |  |  |  |  |  |  |  |  |  |  | 30.45 | 33.25 |
| 527 | PN-541-5p-890 |  |  |  |  |  |  |  | 46.96 |  | 35.39 |  |  |
| 528 | PN-543-3p-264 |  | 38.19 | 48.19 | 29.41 | 38.67 | 39.54 | 28.71 | 45.56 |  |  | 162.46 | 92.12 |
| 529 | PN-543-5p-14352 |  |  |  |  | 27.51 |  |  |  |  |  | 50.87 |  |
| 530 | **PN-551b-3p-28603** | 46.49 | 157.83 | 81.05 | 117.12 | 72.37 | 149.94 | 116.18 | 67.91 | 147.88 | 144.90 | 38.84 | 59.73 |
| 531 | PN-551b-5p-5893 |  |  |  |  |  |  |  |  |  |  |  |  |
| 532 | PN-590-3p-56007 |  |  |  |  |  |  |  |  |  |  |  |  |
| 533 | PN-590-5p-31984 |  |  |  |  |  |  |  |  |  |  |  |  |
| 534 | PN-598-3p-50617 |  |  |  |  | 15.89 |  |  |  |  |  |  |  |
| 535 | **PN-599-5p-187** | 54.66 | 165.67 | 109.74 | 59.69 | 136.56 | 47.96 | 66.35 | 89.67 | 138.10 | 100.88 | 3,850.29 | 899.49 |
| 536 | PN-652-3p-1849 |  |  |  | 34.51 |  |  |  |  |  |  | 85.77 | 100.22 |
| 537 | PN-655-3p-2273 |  |  |  |  |  |  |  |  |  |  |  |  |
| 538 | PN-656-3p-7207 |  |  |  |  |  |  |  |  |  |  |  | 36.83 |
| 539 | PN-656-5p-29663 |  |  |  |  |  |  |  |  |  |  |  |  |
| 540 | PN-660-5p-1115 |  |  |  | 47.56 | 37.72 |  |  |  |  |  | 55.85 | 59.76 |
| 541 | PN-668-5p-28543 |  |  |  |  | 16.88 |  |  |  |  |  | 32.43 |  |
| 542 | **PN-720-5p-9742** | 6,895.52 | 12,517.28 | 6,095.85 | 12,337.71 | 5,606.93 | 11,545.05 | 10,375.53 | 9,715.78 | 6,928.27 | 9,019.19 | 3,132.52 | 2,698.42 |
| 543 | PN-7-3p-8724 |  | 44.35 |  |  | 21.61 |  |  |  |  | 23.20 | 84.66 | 124.75 |
| 544 | PN-758-3p-29208 |  | 101.37 | 58.77 | 40.31 | 79.63 | 57.71 | 137.01 | 90.73 | 91.64 | 60.23 | 37.96 |  |
| 545 | PN-758-5p-11774 |  |  |  |  | 20.68 |  |  |  |  |  | 78.34 | 57.76 |
| 546 | PN-7-5p-18300 |  |  |  |  | 24.31 | 31.99 |  |  |  |  | 370.98 | 283.12 |
| 547 | **PN-7a-5p-541** | 136.12 | 228.73 | 513.81 | 230.06 | 410.22 | 302.49 | 119.27 | 208.22 | 613.04 | 451.06 | 14,416.11 | 17,569.45 |
| 548 | PN-7e-5p-4024 |  |  |  |  |  |  |  |  |  |  | 5,632.06 | 4,856.08 |
| 549 | **PN-875-3p-15533** | 51.51 | 132.87 | 147.05 | 197.85 | 32.92 | 64.82 | 35.15 | 108.82 | 179.06 | 190.93 | 90.28 | 45.61 |
| 550 | PN-875-5p-9827 | 110.08 | 153.77 | 44.30 | 99.82 | 38.02 |  | 93.22 | 89.24 |  | 38.26 |  |  |
| 551 | **PN-92a-3p-3446** | 169.02 | 499.58 | 678.72 | 366.47 | 639.93 | 357.22 | 201.49 | 443.62 | 817.04 | 506.29 | 7,146.86 | 5,510.03 |
| 552 | **PN-92b-3p-50709** | 201.82 | 555.29 | 736.37 | 387.58 | 728.65 | 376.86 | 226.90 | 437.52 | 873.66 | 576.65 | 7,399.75 | 5,799.80 |
| 553 | PN-93-3p-1604 | 20.40 |  | 50.91 | 48.15 | 33.48 | 43.28 |  | 42.38 | 47.67 | 50.95 | 79.63 | 76.50 |
| 554 | PN-93-5p-107 | 102.22 | 206.60 | 238.85 | 159.29 | 388.51 | 133.92 | 69.61 | 153.88 | 226.27 | 230.64 | 1,157.21 | 944.30 |
| 555 | ssc-let-7a | 64.55 | 140.31 | 322.06 | 147.35 | 270.59 | 167.65 | 57.91 | 113.42 | 376.92 | 269.47 | 12,667.80 | 14,503.95 |
| 556 | ssc-let-7c |  | 89.98 | 213.23 | 79.01 | 168.58 | 98.19 | 34.25 | 106.50 | 223.11 | 167.71 | 8,843.54 | 9,660.73 |
| 557 | ssc-let-7e |  | 67.34 | 107.52 | 59.02 | 138.12 | 65.60 |  | 65.71 | 106.67 | 63.73 | 5,312.17 | 4,005.16 |
| 558 | ssc-let-7f |  | 41.14 | 105.61 | 52.53 | 111.22 | 63.01 | 28.09 | 51.44 | 143.20 | 119.90 | 10,383.26 | 11,957.35 |
| 559 | ssc-let-7g |  | 66.71 | 119.15 | 61.64 | 139.67 | 81.94 |  | 58.13 | 173.56 | 117.05 | 5,990.67 | 6,776.61 |
| 560 | **ssc-let-7i** | 201.38 | 178.03 | 386.45 | 288.25 | 387.76 | 145.37 | 177.64 | 110.01 | 407.16 | 420.19 | 8,598.37 | 9,135.91 |
| 561 | ssc-miR-1 |  |  |  |  |  |  |  |  |  |  |  | 37.90 |
| 562 | **ssc-miR-100** | 43.68 | 195.96 | 196.52 | 74.41 | 308.60 | 93.59 | 54.38 | 80.46 | 267.31 | 114.91 | 2,292.28 | 2,196.21 |
| 563 | ssc-miR-101 |  |  |  | 30.16 | 26.84 |  |  |  |  |  |  |  |
| 564 | **ssc-miR-103** | 1,007.62 | 1,770.48 | 2,120.38 | 1,805.13 | 1,700.40 | 1,592.12 | 729.39 | 1,693.51 | 1,481.30 | 1,213.09 | 2,238.59 | 2,283.14 |
| 565 | ssc-miR-105-1 |  |  |  | 29.92 |  |  |  |  |  |  |  |  |
| 566 | ssc-miR-105-2 |  |  |  |  |  |  |  |  |  |  |  |  |
| 567 | **ssc-miR-106a** | 208.19 | 346.84 | 369.66 | 350.72 | 829.51 | 161.75 | 113.08 | 207.55 | 262.59 | 252.49 | 1,272.33 | 901.44 |
| 568 | **ssc-miR-107** | 984.56 | 1,794.01 | 2,105.28 | 1,760.84 | 1,601.89 | 1,606.37 | 731.99 | 1,647.08 | 1,412.78 | 1,194.97 | 2,069.90 | 2,155.99 |
| 569 | ssc-miR-10a |  |  |  |  |  |  |  |  |  |  |  |  |
| 570 | ssc-miR-10b |  |  |  |  |  |  |  |  |  |  |  |  |
| 571 | ssc-miR-122 |  |  |  |  |  |  |  |  |  |  |  |  |
| 572 | ssc-miR-124a |  |  |  | 49.91 |  |  |  |  |  |  | 53.61 |  |
| 573 | **ssc-miR-125a** | 356.67 | 667.14 | 1,020.11 | 674.68 | 1,081.23 | 598.98 | 357.73 | 586.38 | 1,125.11 | 892.08 | 9,076.50 | 9,112.13 |
| 574 | **ssc-miR-125b** | 2,302.28 | 4,788.75 | 6,116.13 | 3,573.44 | 7,823.19 | 3,181.66 | 5,677.14 | 2,862.50 | 7,455.09 | 4,079.58 | 19,163.91 | 17,952.58 |
| 575 | **ssc-miR-127** | 488.32 | 593.88 | 688.65 | 1,131.56 | 518.75 | 527.94 | 1,459.20 | 1,424.25 | 1,906.10 | 2,417.64 | 1,538.50 | 968.79 |
| 576 | ssc-miR-1277 |  |  |  |  |  |  |  |  |  |  |  |  |
| 577 | ssc-miR-128 |  | 89.27 | 72.09 | 47.36 | 98.87 | 64.71 | 36.58 | 80.59 | 93.01 | 80.31 | 1,486.75 | 1,240.86 |
| 578 | **ssc-miR-1285** | 1,403.03 | 2,230.70 | 2,071.75 | 1,948.50 | 2,150.51 | 4,982.91 | 2,226.89 | 2,198.88 | 2,497.70 | 2,164.26 | 2,646.56 | 1,492.73 |
| 579 | ssc-miR-129 |  |  |  |  | 20.72 |  |  |  |  |  |  |  |
| 580 | ssc-miR-1306-3p |  |  |  | 40.60 | 26.02 |  | 29.97 |  |  | 69.05 | 120.87 | 91.73 |
| 581 | **ssc-miR-1306-5p** | 62.67 | 80.56 | 129.56 | 119.28 | 130.44 | 92.41 | 37.00 | 114.97 | 85.64 | 113.88 | 682.20 | 276.99 |
| 582 | **ssc-miR-1307** | 116.45 | 130.69 | 133.13 | 172.66 | 156.57 | 97.00 | 42.86 | 123.40 | 97.04 | 141.46 | 261.67 | 164.65 |
| 583 | **ssc-miR-1308** | 17,272.58 | 11,381.72 | 22,511.09 | 21,165.50 | 20,466.77 | 16,415.72 | 15,414.10 | 12,922.07 | 13,844.29 | 8,741.26 | 9,538.18 | 19,651.32 |
| 584 | **ssc-miR-130a** | 4,361.46 | 3,750.18 | 3,771.36 | 8,579.93 | 468.88 | 4,703.39 | 4,408.25 | 6,592.96 | 8,185.19 | 12,852.28 | 665.72 | 796.62 |
| 585 | **ssc-miR-130b** | 665.52 | 898.13 | 814.52 | 1,412.86 | 267.28 | 872.60 | 423.02 | 719.54 | 766.89 | 1,401.65 | 604.87 | 552.17 |
| 586 | **ssc-miR-133a** | 47.27 | 47.77 | 74.34 | 47.47 | 304.42 | 52.94 | 659.08 | 746.18 | 732.89 | 35.26 | 55.06 | 76.58 |
| 587 | ssc-miR-133a* |  |  |  |  | 17.85 |  |  |  |  |  |  |  |
| 588 | **ssc-miR-133b** | 69.52 | 69.03 | 88.36 | 39.33 | 395.05 | 67.64 | 897.80 | 1,031.88 | 994.03 | 23.50 | 57.28 | 77.59 |
| 589 | ssc-miR-135 |  |  |  |  |  |  |  |  |  |  |  |  |
| 590 | ssc-miR-136 |  |  |  |  |  |  |  |  |  |  |  |  |
| 591 | ssc-miR-139-5p |  |  |  |  |  |  |  |  |  | 39.04 | 191.11 | 332.49 |
| 592 | ssc-miR-140 |  |  |  |  |  |  |  |  |  |  |  |  |
| 593 | **ssc-miR-140*** | 1,166.85 | 2,797.83 | 4,946.67 | 2,761.31 | 357.11 | 4,128.76 | 830.33 | 2,762.18 | 3,194.09 | 4,702.74 | 281.35 | 407.84 |
| 594 | ssc-miR-142 |  |  |  |  |  |  |  |  |  |  |  |  |
| 595 | **ssc-miR-143** | 79.74 | 536.29 | 184.49 | 169.02 | 353.58 | 233.77 | 145.78 | 135.97 | 151.41 | 102.61 | 221.40 | 256.42 |
| 596 | **ssc-miR-145** | 349.25 | 1,115.94 | 822.61 | 558.48 | 630.01 | 896.06 | 398.20 | 809.14 | 951.03 | 889.78 | 1,223.55 | 1,173.25 |
| 597 | ssc-miR-146b |  |  |  |  |  |  |  |  |  |  | 54.29 | 31.85 |
| 598 | ssc-miR-148a |  |  |  | 35.62 | 22.54 |  |  |  |  | 39.56 | 197.15 | 323.00 |
| 599 | ssc-miR-148b |  |  |  | 33.17 | 19.00 |  |  |  |  | 47.63 | 77.90 | 120.44 |
| 600 | ssc-miR-151-3p | 30.71 | 70.20 | 50.81 | 75.02 | 44.35 | 72.06 |  |  | 71.87 | 84.96 | 535.97 | 528.88 |
| 601 | **ssc-miR-151-5p** | 94.53 | 287.40 | 308.28 | 187.61 | 299.37 | 238.02 | 149.77 | 123.14 | 311.22 | 227.98 | 2,167.00 | 2,221.28 |
| 602 | **ssc-miR-152** | 65.77 | 106.80 | 137.56 | 164.19 | 155.76 | 122.57 | 85.82 | 63.86 | 152.55 | 137.13 | 577.62 | 963.48 |
| 603 | ssc-miR-153 |  |  |  |  |  |  |  |  |  |  |  |  |
| 604 | **ssc-miR-15a** | 301.02 | 146.08 | 171.03 | 624.82 | 28.22 | 392.03 | 116.01 | 470.93 | 491.19 | 633.03 | 114.56 | 152.45 |
| 605 | **ssc-miR-15b** | 43.56 | 131.25 | 215.13 | 99.74 | 154.11 | 110.33 | 42.69 | 55.94 | 246.54 | 304.02 | 4,247.73 | 4,404.47 |
| 606 | **ssc-miR-16** | 12,053.37 | 12,859.44 | 13,255.37 | 16,609.95 | 1,292.67 | 16,182.13 | 10,041.43 | 17,148.77 | 21,873.81 | 25,372.80 | 3,008.66 | 3,587.00 |
| 607 | ssc-miR-17-3p |  |  |  | 45.64 | 21.83 | 48.45 |  |  | 46.17 | 82.31 | 37.29 |  |
| 608 | **ssc-miR-17-5p** | 284.18 | 564.58 | 517.34 | 467.30 | 1,209.56 | 221.08 | 143.78 | 273.37 | 339.62 | 341.59 | 1,732.52 | 1,288.52 |
| 609 | ssc-miR-18 |  | 40.09 |  |  | 37.19 |  |  |  |  |  | 37.53 | 33.39 |
| 610 | **ssc-miR-181a** | 919.00 | 2,153.10 | 3,884.84 | 2,638.11 | 5,083.48 | 5,963.08 | 1,614.25 | 1,662.70 | 3,170.06 | 2,661.38 | 4,328.54 | 6,570.38 |
| 611 | **ssc-miR-181b** | 215.42 | 409.10 | 996.66 | 519.30 | 1,288.82 | 1,100.78 | 215.70 | 252.10 | 650.14 | 533.79 | 3,266.75 | 3,966.00 |
| 612 | **ssc-miR-181c** | 223.96 | 409.90 | 436.33 | 738.75 | 1,137.49 | 906.09 | 395.23 | 165.07 | 302.90 | 632.79 | 541.00 | 984.18 |
| 613 | ssc-miR-181d | 34.05 | 39.02 | 83.34 | 80.27 | 245.33 | 136.85 | 39.70 |  | 92.81 | 75.45 | 1,174.33 | 1,135.09 |
| 614 | ssc-miR-183 |  |  |  | 45.62 |  |  |  |  | 38.18 | 51.54 | 1,194.67 | 804.26 |
| 615 | ssc-miR-1839 |  |  |  |  |  |  |  |  |  |  |  |  |
| 616 | ssc-miR-184 |  |  |  |  |  |  |  |  |  |  |  |  |
| 617 | **ssc-miR-185** | 41.77 | 195.04 | 167.89 | 139.35 | 127.25 | 231.77 | 55.65 | 82.23 | 134.77 | 86.20 | 358.93 | 361.87 |
| 618 | ssc-miR-186 |  |  |  |  | 25.17 |  |  |  |  |  | 46.13 | 65.61 |
| 619 | **ssc-miR-191** | 116.44 | 449.58 | 542.04 | 233.23 | 450.72 | 380.07 | 132.63 | 340.30 | 455.60 | 347.88 | 3,816.67 | 3,758.54 |
| 620 | ssc-miR-192 |  | 23.38 |  | 39.06 |  |  |  |  |  |  | 96.55 | 786.66 |
| 621 | **ssc-miR-193a-3p** | 65.72 | 96.27 | 130.58 | 280.83 | 43.46 | 334.19 | 97.50 | 101.39 | 233.91 | 262.19 | 98.04 | 95.54 |
| 622 | ssc-miR-193a-5p | 28.58 | 35.50 |  | 47.37 | 39.36 | 41.05 | 22.31 | 40.70 |  | 46.05 | 104.76 | 91.37 |
| 623 | **ssc-miR-195** | 2,669.07 | 1,334.23 | 1,635.24 | 3,999.19 | 57.17 | 3,332.44 | 1,581.50 | 1,944.01 | 4,128.95 | 3,755.70 | 258.82 | 306.72 |
| 624 | ssc-miR-196a |  |  |  | 30.89 |  |  |  |  |  |  |  |  |
| 625 | ssc-miR-196b |  |  |  |  | 15.93 |  |  |  |  |  |  |  |
| 626 | **ssc-miR-199a-3p** | 530.85 | 1,665.99 | 1,536.03 | 932.11 | 1,633.86 | 703.73 | 588.12 | 954.10 | 1,736.16 | 965.99 | 10,985.70 | 11,435.12 |
| 627 | **ssc-miR-199a-5p** | 1,279.48 | 2,537.73 | 2,176.01 | 1,731.51 | 3,277.58 | 1,645.78 | 1,101.24 | 2,817.38 | 2,003.95 | 1,554.12 | 1,680.86 | 1,676.87 |
| 628 | **ssc-miR-199b*** | 519.89 | 767.30 | 859.09 | 799.32 | 1,072.92 | 270.07 | 535.46 | 288.19 | 974.69 | 770.95 | 11,337.80 | 11,703.19 |
| 629 | ssc-miR-19a | 36.78 |  |  | 31.37 | 273.61 |  | 37.45 |  |  |  |  |  |
| 630 | **ssc-miR-19b** | 1,732.78 | 1,620.49 | 771.89 | 2,060.26 | 4,093.57 | 263.23 | 1,656.64 | 650.25 | 362.44 | 533.49 | 112.50 | 104.76 |
| 631 | **ssc-miR-20** | 81.76 | 125.77 | 112.83 | 104.71 | 331.85 | 64.10 | 49.42 | 81.85 | 92.25 | 84.04 | 668.06 | 516.91 |
| 632 | ssc-miR-202 |  |  |  |  |  |  |  |  |  |  |  |  |
| 633 | ssc-miR-204 |  |  |  |  |  |  |  |  |  |  | 26.63 |  |
| 634 | **ssc-miR-205** | 1,663.58 | 1,345.66 | 3,963.20 | 1,984.99 | 4,339.49 | 1,819.66 | 1,130.88 | 1,913.24 | 2,487.51 | 2,609.17 | 5,956.77 | 8,671.07 |
| 635 | ssc-miR-206 |  |  |  | 43.53 | 67.13 |  | 67.69 |  | 161.49 |  | 311.25 | 380.63 |
| 636 | ssc-miR-208b |  |  |  |  |  |  |  |  |  |  |  |  |
| 637 | ssc-miR-21 |  |  |  |  | 19.19 |  |  |  |  |  | 630.24 | 547.54 |
| 638 | **ssc-miR-210** | 233.79 | 263.47 | 413.54 | 667.77 | 40.98 | 647.45 | 208.36 | 457.50 | 949.99 | 1,076.31 | 141.38 | 394.46 |
| 639 | **ssc-miR-214** | 3,899.05 | 6,432.82 | 8,966.32 | 5,778.22 | 12,851.45 | 4,181.78 | 5,638.21 | 8,867.14 | 8,148.71 | 6,020.13 | 20,293.06 | 13,210.47 |
| 640 | ssc-miR-215 |  |  |  |  |  |  |  |  |  |  | 49.55 | 52.33 |
| 641 | ssc-miR-216 |  |  |  |  |  |  |  |  |  |  | 34.51 |  |
| 642 | ssc-miR-217 |  |  |  |  |  |  |  |  |  |  |  |  |
| 643 | **ssc-miR-221** | 250.52 | 205.45 | 406.95 | 488.22 | 186.47 | 285.67 | 165.35 | 212.44 | 389.08 | 722.41 | 581.72 | 537.56 |
| 644 | **ssc-miR-222** | 138.86 | 162.03 | 177.40 | 136.28 | 158.66 | 122.79 | 45.37 | 131.76 | 141.25 | 212.06 | 728.19 | 544.01 |
| 645 | ssc-miR-22-3p |  |  | 50.88 | 37.95 | 31.79 | 122.38 |  |  |  |  | 54.00 | 111.03 |
| 646 | ssc-miR-224 | 27.02 |  |  | 54.69 | 20.34 |  |  |  |  |  | 124.18 |  |
| 647 | ssc-miR-22-5p |  |  |  |  |  |  |  |  |  |  | 37.06 |  |
| 648 | **ssc-miR-23a** | 260.27 | 415.27 | 654.90 | 432.59 | 551.67 | 274.97 | 144.43 | 226.66 | 522.51 | 409.87 | 9,469.62 | 11,512.73 |
| 649 | **ssc-miR-23b** | 425.38 | 849.57 | 1,202.91 | 666.00 | 839.98 | 423.11 | 283.06 | 417.75 | 817.96 | 715.32 | 10,562.62 | 13,032.66 |
| 650 | **ssc-miR-24** | 622.87 | 1,288.81 | 1,346.15 | 867.34 | 1,394.58 | 677.34 | 502.49 | 740.90 | 935.38 | 651.47 | 4,103.43 | 4,322.49 |
| 651 | ssc-miR-24* |  |  |  |  |  |  |  |  |  |  |  |  |
| 652 | **ssc-miR-26a** | 491.54 | 868.26 | 1,100.04 | 787.32 | 1,063.46 | 533.29 | 472.09 | 480.80 | 1,136.24 | 837.02 | 15,087.24 | 21,559.94 |
| 653 | **ssc-miR-27a** | 49.59 | 82.78 | 88.48 | 69.17 | 112.20 | 61.75 | 25.58 | 65.01 | 70.08 | 68.88 | 1,768.62 | 1,573.41 |
| 654 | **ssc-miR-27b** | 246.58 | 331.92 | 456.08 | 311.81 | 269.45 | 184.90 | 126.65 | 130.73 | 296.64 | 298.90 | 3,161.91 | 3,880.22 |
| 655 | ssc-miR-27b* |  |  |  |  | 26.41 |  |  |  |  |  | 71.50 | 68.77 |
| 656 | ssc-miR-28-3p | 39.25 | 50.01 | 40.49 | 34.12 | 34.70 | 49.11 | 59.13 |  |  | 74.17 | 217.67 | 290.84 |
| 657 | ssc-miR-28-5p |  |  |  |  |  |  |  |  |  |  | 49.36 | 39.19 |
| 658 | ssc-miR-299 |  | 56.58 | 75.67 | 34.99 | 90.33 | 51.85 | 67.03 | 95.19 | 118.77 | 59.32 | 376.07 | 314.84 |
| 659 | ssc-miR-29a |  |  |  |  |  |  |  |  |  |  | 40.19 | 28.59 |
| 660 | ssc-miR-29b |  |  |  |  |  |  |  |  |  |  |  |  |
| 661 | ssc-miR-29c |  |  |  |  |  |  |  |  |  |  |  |  |
| 662 | ssc-miR-301 |  |  |  |  | 16.92 |  |  |  |  |  |  |  |
| 663 | ssc-miR-30a-3p |  |  |  |  |  |  |  |  |  |  | 61.19 | 59.75 |
| 664 | **ssc-miR-30a-5p** | 44.24 | 92.15 | 87.54 | 63.00 | 116.93 | 71.60 | 80.64 | 62.96 | 91.08 | 73.75 | 348.62 | 476.47 |
| 665 | ssc-miR-30b-3p |  |  |  |  | 18.79 |  |  |  |  |  | 58.85 |  |
| 666 | **ssc-miR-30b-5p** | 201.28 | 252.60 | 403.04 | 322.87 | 431.00 | 216.20 | 156.44 | 129.67 | 260.24 | 193.28 | 1,661.93 | 1,728.08 |
| 667 | **ssc-miR-30c** | 124.09 | 246.53 | 354.23 | 198.55 | 299.61 | 196.18 | 93.51 | 162.16 | 328.72 | 249.55 | 2,209.36 | 2,316.26 |
| 668 | **ssc-miR-30d** | 205.52 | 498.75 | 513.22 | 394.55 | 459.15 | 407.05 | 289.65 | 340.18 | 528.24 | 368.17 | 1,872.83 | 2,277.36 |
| 669 | ssc-miR-30e-3p |  |  |  |  |  |  |  |  |  |  | 46.14 | 58.10 |
| 670 | **ssc-miR-30e-5p** | 177.47 | 440.46 | 317.75 | 376.91 | 487.32 | 286.83 | 280.09 | 241.32 | 318.16 | 239.30 | 550.02 | 611.37 |
| 671 | ssc-miR-32 |  |  |  |  |  |  |  |  |  |  |  |  |
| 672 | **ssc-miR-320** | 591.03 | 747.40 | 1,538.74 | 1,321.05 | 1,211.05 | 957.77 | 380.11 | 1,464.85 | 1,315.03 | 1,992.99 | 4,198.69 | 3,178.54 |
| 673 | ssc-miR-323 |  | 34.42 | 50.93 | 49.61 | 43.54 | 30.95 |  | 50.87 | 69.08 | 38.55 | 222.83 | 136.44 |
| 674 | **ssc-miR-324** | 24.51 | 57.02 | 66.74 | 46.11 | 105.22 | 65.82 | 37.65 | 53.28 | 52.13 | 75.58 | 163.40 | 169.09 |
| 675 | ssc-miR-325 |  |  |  |  |  |  |  |  |  |  |  |  |
| 676 | ssc-miR-326 |  | 33.53 | 55.10 | 35.09 |  | 57.43 |  |  | 49.67 | 87.87 | 54.43 | 43.38 |
| 677 | ssc-miR-328 | 18.43 |  | 50.61 | 46.73 | 23.98 | 49.62 |  | 46.45 | 29.93 | 51.36 | 105.61 | 127.71 |
| 678 | **ssc-miR-331-3p** | 473.34 | 898.94 | 681.43 | 954.19 | 365.47 | 743.44 | 630.65 | 490.23 | 590.15 | 814.05 | 282.16 | 293.31 |
| 679 | ssc-miR-331-5p |  |  |  |  | 17.64 |  |  |  |  |  | 33.63 | 31.58 |
| 680 | ssc-miR-335 |  |  |  |  |  |  |  |  |  |  | 57.60 | 270.94 |
| 681 | ssc-miR-338 |  |  |  |  |  |  |  |  |  |  |  |  |
| 682 | **ssc-miR-339** | 240.57 | 567.14 | 524.39 | 565.47 | 150.97 | 851.07 | 402.47 | 776.49 | 1,124.72 | 1,535.96 | 124.69 | 189.87 |
| 683 | ssc-miR-340 |  |  |  |  |  |  |  |  |  |  |  |  |
| 684 | **ssc-miR-342** | 37.55 | 137.13 | 138.12 | 88.72 | 118.41 | 112.81 | 26.68 | 66.75 | 133.78 | 127.09 | 1,004.75 | 797.99 |
| 685 | ssc-miR-345-3p |  |  |  | 65.19 | 21.65 |  |  |  |  | 41.87 | 45.64 | 56.18 |
| 686 | ssc-miR-345-5p |  | 52.15 | 63.28 | 149.11 |  | 85.90 | 51.33 | 58.74 | 252.74 | 526.48 | 36.25 |  |
| 687 | ssc-miR-34a |  |  |  |  | 18.84 |  |  |  |  |  | 33.16 |  |
| 688 | ssc-miR-34c |  |  |  | 51.91 |  |  |  |  |  | 46.00 |  |  |
| 689 | ssc-miR-361-3p |  |  |  | 38.38 | 16.89 | 62.59 |  |  |  | 40.54 | 39.32 | 52.01 |
| 690 | **ssc-miR-361-5p** | 44.71 | 126.53 | 146.67 | 105.27 | 139.07 | 92.20 | 51.77 | 71.68 | 271.91 | 167.55 | 2,225.49 | 2,013.07 |
| 691 | ssc-miR-363 |  |  |  | 30.81 |  |  |  |  |  | 30.74 | 42.91 |  |
| 692 | ssc-miR-365 |  |  |  |  |  |  |  |  |  | 21.10 | 83.97 | 52.13 |
| 693 | ssc-miR-369 |  |  |  |  |  |  |  |  |  |  |  |  |
| 694 | ssc-miR-374a |  |  |  |  |  |  |  |  |  |  |  |  |
| 695 | ssc-miR-374a* |  |  |  |  |  |  |  |  |  |  |  |  |
| 696 | ssc-miR-374b |  |  |  |  |  |  |  |  |  |  | 69.57 | 59.68 |
| 697 | ssc-miR-376a |  |  |  |  |  |  | 72.15 |  |  |  |  |  |
| 698 | ssc-miR-376a* |  |  |  | 36.54 | 31.43 | 38.89 | 63.52 |  |  |  |  |  |
| 699 | **ssc-miR-378** | 53.12 | 88.71 | 141.12 | 110.09 | 218.76 | 131.92 | 123.69 | 137.13 | 146.96 | 82.97 | 168.36 | 157.44 |
| 700 | ssc-miR-423-3p |  |  |  |  | 17.79 |  |  |  |  |  | 54.37 | 58.94 |
| 701 | **ssc-miR-423-5p** | 224.26 | 224.16 | 393.93 | 380.75 | 539.62 | 213.56 | 200.96 | 204.73 | 252.62 | 386.01 | 1,837.60 | 929.55 |
| 702 | ssc-miR-424 |  |  |  |  | 17.03 |  |  |  |  |  |  |  |
| 703 | ssc-miR-424* |  |  |  | 29.97 |  | 26.67 |  |  |  |  | 44.72 | 54.07 |
| 704 | ssc-miR-425-3p |  |  | 44.60 | 54.23 | 22.45 | 57.08 |  | 49.41 |  | 71.79 | 38.57 | 39.15 |
| 705 | **ssc-miR-425-5p** | 19.77 | 63.78 | 82.43 | 44.59 | 87.40 | 91.87 | 21.82 | 55.09 | 83.72 | 61.73 | 287.96 | 309.76 |
| 706 | ssc-miR-432 |  |  |  |  | 33.29 |  |  |  | 24.46 |  | 275.22 | 126.44 |
| 707 | ssc-miR-450a |  |  |  |  |  |  |  |  |  |  |  |  |
| 708 | ssc-miR-450b |  |  |  |  |  |  |  |  |  |  |  |  |
| 709 | ssc-miR-450c |  |  |  | 32.53 | 21.07 |  |  |  |  |  |  |  |
| 710 | ssc-miR-450c* |  |  |  |  |  |  |  |  |  |  |  |  |
| 711 | **ssc-miR-455** | 727.32 | 1,786.39 | 1,531.10 | 1,609.83 | 816.08 | 1,676.41 | 764.72 | 1,479.27 | 1,350.76 | 1,986.34 | 899.09 | 930.29 |
| 712 | ssc-miR-486 |  |  |  |  |  |  |  |  |  |  | 55.92 | 34.00 |
| 713 | **ssc-miR-497** | 39.08 | 107.89 | 92.62 | 63.26 | 28.22 | 108.31 | 39.25 | 71.69 | 118.49 | 105.20 | 39.68 | 51.37 |
| 714 | ssc-miR-499 |  |  |  |  |  |  |  |  |  |  |  |  |
| 715 | ssc-miR-500 |  |  |  |  |  |  |  |  |  |  | 57.68 | 44.86 |
| 716 | ssc-miR-503 |  |  |  |  | 21.89 |  |  |  |  |  | 29.76 |  |
| 717 | **ssc-miR-504** |  | 41.49 | 50.96 | 133.53 | 26.93 | 84.49 | 121.00 | 130.44 | 425.27 | 731.74 | 115.52 | 105.79 |
| 718 | ssc-miR-505 |  | 52.40 | 67.49 | 58.71 | 58.79 | 49.31 |  |  | 79.02 | 91.19 | 513.70 | 430.49 |
| 719 | ssc-miR-532-3p |  |  | 47.23 | 54.26 | 28.33 |  | 35.61 |  | 59.14 | 61.25 | 156.06 | 136.73 |
| 720 | ssc-miR-532-5p |  |  |  | 32.39 | 39.04 | 32.52 |  |  | 31.64 |  | 124.48 | 132.06 |
| 721 | ssc-miR-542-3p |  |  |  |  |  |  |  |  |  |  |  |  |
| 722 | ssc-miR-542-5p |  |  |  |  |  |  |  | 64.21 | 50.86 |  |  |  |
| 723 | **ssc-miR-574** | 8,568.92 | 5,577.42 | 5,920.71 | 12,599.55 | 1,380.87 | 1,134.35 | 8,364.59 | 4,349.22 | 3,736.88 | 10,341.48 | 2,524.24 | 3,655.42 |
| 724 | ssc-miR-628 |  |  |  |  |  |  |  |  |  |  | 38.01 | 49.77 |
| 725 | ssc-miR-664-3p |  |  |  | 39.05 | 22.71 |  |  |  |  |  | 42.07 | 49.19 |
| 726 | ssc-miR-664-5p |  |  |  |  |  |  |  |  |  |  | 37.33 | 35.84 |
| 727 | ssc-miR-676 |  | 52.86 | 70.21 | 39.06 | 87.45 | 39.41 |  | 57.47 | 60.75 | 69.54 | 389.35 | 318.07 |
| 728 | ssc-miR-7 |  |  |  |  |  |  |  |  |  |  | 110.53 | 86.88 |
| 729 | ssc-miR-708 | 29.73 | 56.80 | 53.51 | 44.93 | 71.83 |  |  | 44.25 |  | 36.49 | 231.52 | 251.88 |
| 730 | **ssc-miR-744** | 134.16 | 216.74 | 282.10 | 448.49 | 48.22 | 340.95 | 118.25 | 373.85 | 513.21 | 833.53 | 257.15 | 222.53 |
| 731 | **ssc-miR-758** | 33.85 | 85.58 | 64.62 | 59.07 | 76.02 | 51.63 | 129.26 | 83.79 | 92.71 | 64.19 | 57.59 | 48.06 |
| 732 | ssc-miR-769 |  |  |  |  |  |  |  |  |  | 63.06 | 45.80 | 38.25 |
| 733 | ssc-miR-885-3p |  |  |  |  |  |  |  |  |  |  | 58.30 |  |
| 734 | ssc-miR-885-5p |  |  |  |  |  |  |  |  |  |  | 35.73 |  |
| 735 | ssc-miR-9-1 |  |  |  |  |  |  |  |  |  |  | 140.53 |  |
| 736 | **ssc-miR-92a** | 237.50 | 575.17 | 834.88 | 499.03 | 808.96 | 400.68 | 287.14 | 493.53 | 996.35 | 692.02 | 8,339.26 | 6,613.34 |
| 737 | **ssc-miR-92b** | 69.74 | 108.81 | 202.77 | 177.93 | 166.71 | 130.05 | 75.56 | 138.96 | 252.13 | 286.55 | 3,389.30 | 2,434.80 |
| 738 | ssc-miR-935 |  |  |  |  |  |  |  |  |  |  | 50.08 | 33.79 |
| 739 | ssc-miR-95 |  |  |  |  |  |  |  |  |  |  |  |  |
| 740 | ssc-miR-98 |  |  |  |  |  |  |  |  |  |  | 1,575.40 | 1,442.85 |
| 741 | **ssc-miR-99a** | 137.71 | 443.38 | 602.58 | 297.90 | 492.15 | 304.05 | 336.49 | 231.27 | 623.17 | 278.89 | 1,994.37 | 2,441.55 |
| 742 | **ssc-miR-99b** | 91.77 | 348.83 | 319.18 | 260.92 | 350.33 | 245.43 | 90.29 | 226.13 | 344.53 | 334.64 | 2,265.26 | 1,787.46 |
