# Supplementary material for: Identification of differential microRNA expression during tooth morphogenesis in the heterodont dentition of miniature pigs, SusScrofa
Source: BMC Dev Biol. 2015 Dec 29;15:51. doi: 10.1186/s12861-015-0099-0 (PMC4696248; doi:10.1186/s12861-015-0099-0)
Supplement: Additional file 5: — Statistic Test and Clustering Analysis of key microRNA. (DOC 108 kb) [file 12861_2015_99_MOESM5_ESM.doc]

**Additional file 5 Statistic Test and Clustering Analysis of key microRNA**

| **Test Name** | **No.** | **Reporter Name** | **p-value** | **Di(Mean)** | **Dc(Mean)** | **Dpm(Mean)** | **Dm(Mean)** |
| --- | --- | --- | --- | --- | --- | --- | --- |
| Di vs Dc vs Dpm vs Dm (p-value < 0.01) | 28 | miR-133a-3p-1466 | 0.000274 | 58 | 146 | 904 | 23 |
|  | 588 | ssc-miR-133b | 0.000396 | 72 | 214 | 1,359 | 23 |
|  | 586 | ssc-miR-133a | 0.000594 | 57 | 165 | 1,000 | 25 |
|  | 29 | miR-133b-3p-56920 | 0.00108 | 59 | 127 | 707 | 15 |
|  | 399 | PN-103-3p-30235 | 0.0013 | 1,789 | 1,527 | 1,237 | 686 |
|  | 432 | PN-1937b-5p-17039 | 0.00215 | 27,272 | 25,692 | 23,242 | 9,060 |
|  | 568 | ssc-miR-107 | 0.0029 | 2,190 | 1,886 | 1,716 | 809 |
|  | 564 | ssc-miR-103 | 0.00326 | 2,195 | 1,929 | 1,758 | 852 |
|  | 195 | PC-103-5p-40816 | 0.00387 | 1,399 | 1,426 | 984 | 706 |
|  | 8 | miR-107-3p-33 | 0.00426 | 2,477 | 2,159 | 2,027 | 1,120 |
|  | 194 | PC-103-3p-49493 | 0.00713 | 2,868 | 2,479 | 2,336 | 1,266 |
|  | Following transcripts are statistically significant but have low signals (signal < 500) | | | | | | |
|  | 670 | ssc-miR-30e-5p | 0.00312 | 436 | 459 | 401 | 176 |
|  | 510 | PN-485-3p-375 | 0.0034 | 171 | 151 | 428 | 91 |
|  | 505 | PN-411-5p-771 | 0.0035 | 351 | 246 | 578 | 30 |
|  | 458 | PN-301a-3p-20657 | 0.00397 | 341 | 335 | 187 | 27 |
|  | 248 | PC-3p-41498 | 0.00837 | 145 | 127 | 125 | 1,312 |
|  | 274 | PC-3p-53437 | 0.00532 | 104 | 81 | 91 | 576 |
|  | 246 | PC-3p-40920 | 0.000813 | 7 | 5 | 8 | 34 |
|  | 599 | ssc-miR-148b | 0.00238 | 25 | 24 | 22 | 44 |
|  | 699 | ssc-miR-378 | 0.00422 | 127 | 184 | 187 | 58 |
|  | 277 | PC-3p-54511 | 0.00621 | 17 | 25 | 20 | 111 |
|  | 501 | PN-409-5p-873 | 0.00952 | 18 | 24 | 34 | 20 |
| Di vs Dc (p-value < 0.05) | Following transcripts are statistically significant but have low signals (signal < 500) | | | | | | |
|  | 654 | ssc-miR-27b | 0.0301 | 485 | 296 | / | / |
| Dc vs Dpm (p-value < 0.05) | 575 | ssc-miR-127 | 0.0189 | / | 814 | 2226 | / |
| Dpm vs Dm (p-value < 0.01) | 588 | ssc-miR-133b | 0.000248 | / | / | 1359 | 23 |
|  | 586 | ssc-miR-133a | 0.000258 | / | / | 1000 | 25 |
|  | 29 | miR-133b-3p-56920 | 0.000377 | / | / | 707 | 15 |
|  | 28 | miR-133a-3p-1466 | 0.000382 | / | / | 904 | 23 |
|  | Following transcripts are statistically significant but have low signals (signal < 500) | | | | | | |
|  | 150 | miR-431-5p-35690 | 0.0066 | / | / | 56 | 13 |
|  | 471 | PN-329a-3p-32943 | 0.00699 | / | / | 32 | 24 |
| Di vs Dpm (p-value < 0.01) | 586 | ssc-miR-133a | 0.000507 | 57 | / | 1000 | / |
|  | 588 | ssc-miR-133b | 0.000568 | 72 | / | 1359 | / |
|  | 575 | ssc-miR-127 | 0.000893 | 832 | / | 2226 | / |
|  | 28 | miR-133a-3p-1466 | 0.00225 | 58 | / | 904 | / |
|  | 29 | miR-133b-3p-56920 | 0.00334 | 59 | / | 707 | / |
|  | Following transcripts are statistically significant but have low signals (signal < 500) | | | | | | |
|  | 513 | PN-487b-3p-443 | 0.00176 | 57 | / | 95 | / |
|  | 106 | miR-27a-3p-1818 | 0.00235 | 304 | / | 177 | / |
|  | 717 | ssc-miR-504 | 0.00985 | 45 | / | 265 | / |
| Dcvs Dm (p-value < 0.01) | Following transcripts are statistically significant but have low signals (signal < 500) | | | | | | |
|  | 246 | PC-3p-40920 | 0.00754 | / | 5 | / | 34 |
| Di vs Dm (p-value < 0.01) | 399 | PN-103-3p-30235 | 0.0055 | 1789 | / | / | 686 |
|  | Following transcripts are statistically significant but have low signals (signal < 500) | | | | | | |
|  | 246 | PC-3p-40920 | 0.00269 | 7 | / | / | 34 |
|  | 588 | ssc-miR-133b | 0.00735 | 72 | / | / | 23 |
|  | 70 | miR-1983-3p-46851 | 0.00749 | 39 | / | / | 16 |
|  | 586 | ssc-miR-133a | 0.00756 | 57 | / | / | 25 |
