# Supplementary figures and images for: Identification of differential microRNA expression during tooth morphogenesis in the heterodont dentition of miniature pigs, SusScrofa
Source: BMC Dev Biol. 2015 Dec 29;15:51. doi: 10.1186/s12861-015-0099-0 (PMC4696248; doi:10.1186/s12861-015-0099-0)

Di

Dc

Dpm

Dm

E40

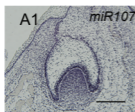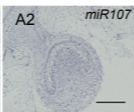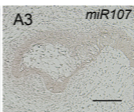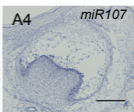

E50

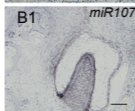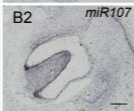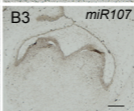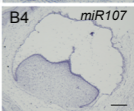

E60

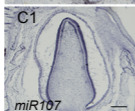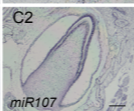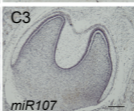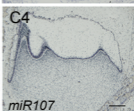

E40

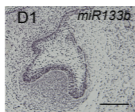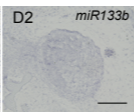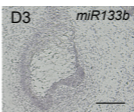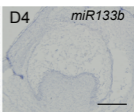

E50

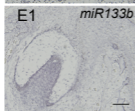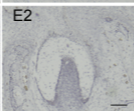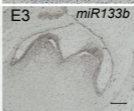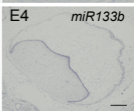

E60

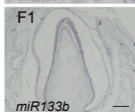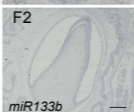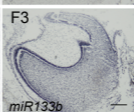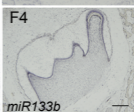

Supplement: Additional file 6: — Expression patterns of miRNAs in the four types of teeth during three tooth developmental stages revealed by in situ hybridization. (A1–C4) At E40, miR-107 was expressed in both the epithelium and mesenchyme of the incisor, canine and molar, but expression in the premolar was not detected by in situ hybridization (A1–A4). At E50, localization of miR-107 in all four types of teeth stayed the same, but the expression level was more restricted in the inner enamel epithelium in the incisor, canine, and molar (B1–B4). At E60, mir-107 expression in the premolar increased significantly; in the premolar as in the other three types of teeth, the location was restricted in the inner enamel epithelium (C1–C4). (D1–F4) At E40, miR-133b was expressed in the both epithelium and mesenchyme of all four types of teeth, with a higher signal in the incisor and a lower signal in the other three types of teeth (D1–D4). At E50, miR-133b expression in all four types of teeth stayed the same, but with a lower signal in the incisor (E1–E4). At E60, expression was more restricted in the inner enamel epithelium and increased expression was found in the premolar and molar (F1–F4). Scale bar, 200 μm. Di, first deciduous incisor; Dc, deciduous canine; Dpm, second deciduous premolar; Dm, deciduous molar; E40, embryonic day 40; E50, embryonic day 50; E60, embryonic day 60. (PDF 4755 kb) [file 12861_2015_99_MOESM6_ESM.pdf]

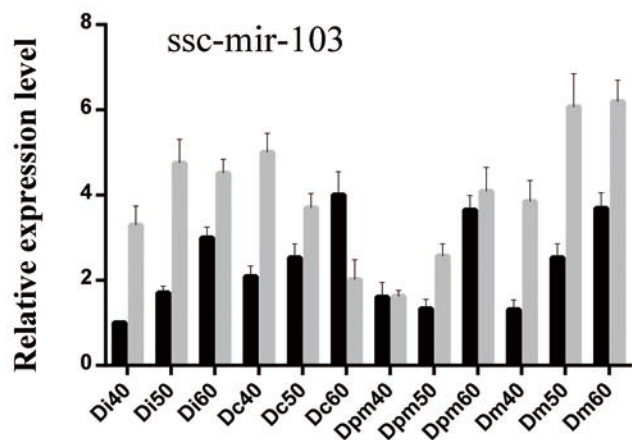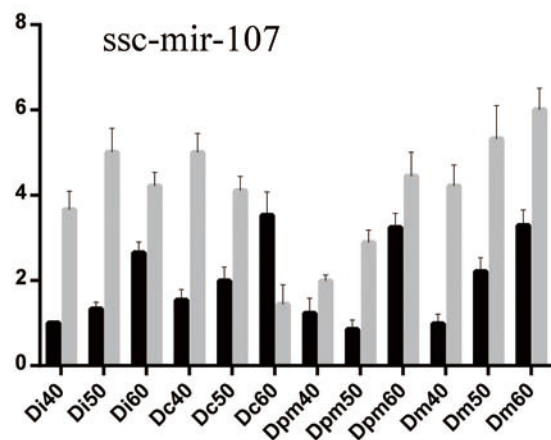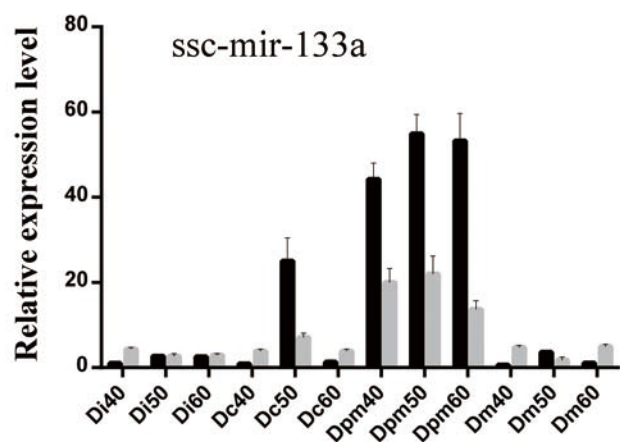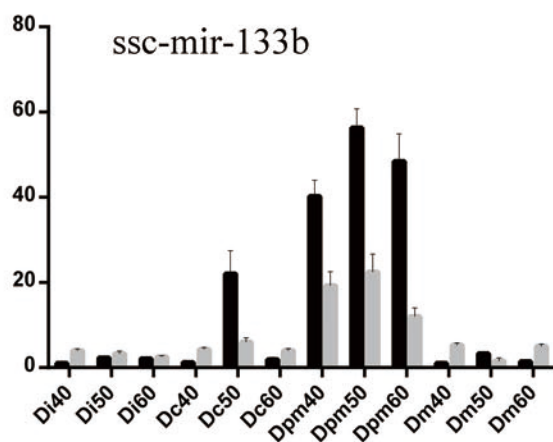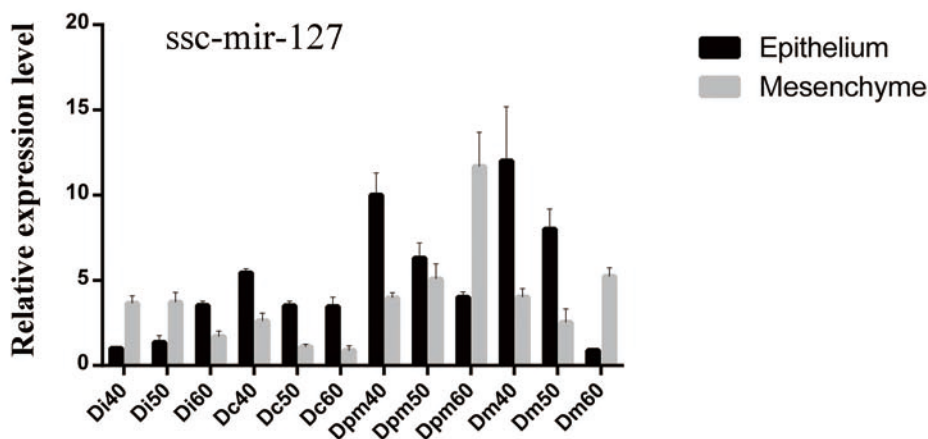

Supplement: Additional file 7: — Validation of differentially expressed miRNAs detection in both epithelium and mesenchyme by real-time RT-PCR. We separated epithelium and mesenchyme from each tooth germ and detected the expression of each miRNA respectively. Each detection was replicated three times and the average is shown with the standard deviation. (PDF 4386 kb) [file 12861_2015_99_MOESM7_ESM.pdf]
